# The co‐existence of multiple oak leaf flushes contributes to the large within‐tree variation in chemistry, insect attack and pathogen infection

**DOI:** 10.1111/nph.18209

**Published:** 2022-05-26

**Authors:** Álvaro Gaytán, Xoaquín Moreira, Bastien Castagneyrol, Inge Van Halder, Pieter De Frenne, Camille Meeussen, Bart G. H. Timmermans, Jan P. J. G. Ten Hoopen, Pil U. Rasmussen, Nick Bos, Raimo Jaatinen, Pertti Pulkkinen, Sara Söderlund, Felisa Covelo, Karl Gotthard, Ayco J. M. Tack

**Affiliations:** ^1^ Department of Ecology, Environment and Plant Sciences Stockholm University Svante Arrhenius väg 20A Stockholm Sweden; ^2^ Misión Biológica de Galicia (MBG‐CSIC) Apdo. 28 36080 Pontevedra, Galicia Spain; ^3^ BIOGECO, INRAE University of Bordeaux 33610 Cestas France; ^4^ Forest & Nature Laboratory Ghent University Geraardsbergsesteenweg 267 BE‐9090 Gontrode‐Melle Belgium; ^5^ Department of Agriculture Louis Bolk Institute Kosterijland 3‐5 3981 AJ Bunnik the Netherlands; ^6^ OneNature Ecology Radarpad 22 6816 TP Arnhem the Netherlands; ^7^ The National Research Centre for the Working Environment 2100 Copenhagen Denmark; ^8^ Section for Ecology & Evolution University of Copenhagen 2200 Copenhagen Denmark; ^9^ Natural Resources Institute Finland, Haapastensyrjä Breeding Station FI‐16200 Läyliäinen Finland; ^10^ Departamento de Sistemas Físicos Químicos y Naturales Universidad Pablo de Olavide Carretera de Utrera km. 1 41013 Seville Spain; ^11^ Department of Zoology Stockholm University Svante Arrhenius väg 18B SE‐106 91 Stockholm Sweden

**Keywords:** herbivory, leaf chemistry, leaf flush, pathogen infection, polycyclism, *Quercus robur*

## Abstract

- Many plant species produce multiple leaf flushes during the growing season, which might have major consequences for within‐plant variation in chemistry and species interactions. Yet, we lack a theoretical or empirical framework for how differences among leaf flushes might shape variation in damage by insects and diseases.
- We assessed the impact of leaf flush identity on leaf chemistry, insect attack and pathogen infection on the pedunculate oak *Quercus robur* by sampling leaves from each leaf flush in 20 populations across seven European countries during an entire growing season.
- The first leaf flush had higher levels of primary compounds, and lower levels of secondary compounds, than the second flush, whereas plant chemistry was highly variable in the third flush. Insect attack decreased from the first to the third flush, whereas infection by oak powdery mildew was lowest on leaves from the first flush. The relationship between plant chemistry, insect attack and pathogen infection varied strongly among leaf flushes and seasons.
- Our findings demonstrate the importance of considering differences among leaf flushes for our understanding of within‐tree variation in chemistry, insect attack and disease levels, something particularly relevant given the expected increase in the number of leaf flushes with climate change.

## Introduction

Leaf flush in spring is one of the most conspicuous events in extratropical regions of the globe. Nevertheless, numerous plant species continuously produce leaves, or produce distinct leaf flushes during the growing season (Auerbach & Simberloff, 1984; Moles & Westoby, 2000; Prado *et al*., 2014). As newly produced leaves might differ from older leaves in nutrient content, secondary chemistry and resistance against pests and pathogens, the temporal pattern of leaf production might contribute to variation in insect attack and disease levels within and among plants (Auerbach & Simberloff, 1984; Call & St. Clair, 2017; Fuenzalida *et al*., 2019). Since the frequency and abundance of multiple leaf flushes is expected to increase with climate change due to higher temperatures and a longer growing season (Soolanayakanahally *et al*., 2013; Hamilton *et al*., 2016), it is important to examine how differences among leaf flushes shape within‐plant and among plant variation in leaf chemistry and species interactions. Yet, very few studies on plant chemistry, insect and pathogen attack distinguish among leaf flushes (but see St. Clair *et al*., 2009; Fuenzalida *et al*., 2019), and we lack a theoretical or empirical framework to predict such changes.

The co‐existence of multiple leaf flushes during a single year (polycyclism) is a common phenomenon in both tropical and temperate forests, and has been observed for tree species, forbs and grasses (Lieberman & Lieberman, 1984; Moles & Westoby, 2000; Battey, 2003; Elliott *et al*., 2006; Prado *et al*., 2014). While some of these plant species produce leaves continuously across the year or season, the production of different leaf flushes is highly synchronized at specific times for other plant species, such as tea, beech and oak, thereby creating the appearance of distinct leaf flushes. The production of new leaf flushes often depends on climatic factors and abiotic or biotic stresses (Hilton *et al*., 1987; Wesołowski & Rowiński, 2008; Fuenzalida *et al*., 2019). Among abiotic factors, Hamilton *et al*. (2016) showed that second and subsequent leaf flushes were more common in white spruce at locations where growing seasons were longer, and Soolanayakanahally *et al*. (2013) found that *Populus balsamifera* was more likely to produce an extra leaf flush when the temperature was high during the latter part of the summer. Among biotic factors, previous studies suggested that the production of the second and subsequent leaf flushes can be a compensatory response to high herbivory rates during the early season (Hilton *et al*., 1987; Bobinac *et al*., 2012; Piper & Fajardo, 2014). Overall, the general expectation is that the number of leaf flushes per year is larger in the warmer part of the distribution of a given plant species. Within a given climate, however, the timing of the second or subsequent leaf flushes might re‐occur at the same time every year. For example, in several European countries, such as Belgium, Germany or the Netherlands, the freshly emerging shoots from the second leaf flush on oak are named after Saint John the Baptist, who is celebrated on 24 June (Kobel, 1954; Lyr *et al*., 1967).

The primary and secondary chemistry of leaves tend to change with the progression of the growing season, when leaves often decrease in nutrient content and increase in secondary chemistry, even though the pattern is variable among and within species (Barton & Koricheva, 2010). Changes within the leaves are affected by seasonal changes in the climate and by biotic factors such as herbivory, but are also due to ontogenetic changes in leaf chemistry (Barton & Koricheva, 2010). It might therefore be expected that plants with multiple leaf flushes simultaneously harbour leaves that strongly differ in the levels of nutrients and secondary chemistry. Indeed, several studies have reported that leaves from the second flush have lower nutrient contents and higher concentrations of secondary compounds (Potter & Redmond, 1989; St. Clair *et al*., 2009; Fuenzalida *et al*., 2019). Yet, this important driver of variation of plant chemistry within plants – and among plants, if individual plants differ in their tendency to produce multiple leaf flushes – is rarely considered when describing spatial and temporal patterns in leaf chemistry.

**Fig. 1:**
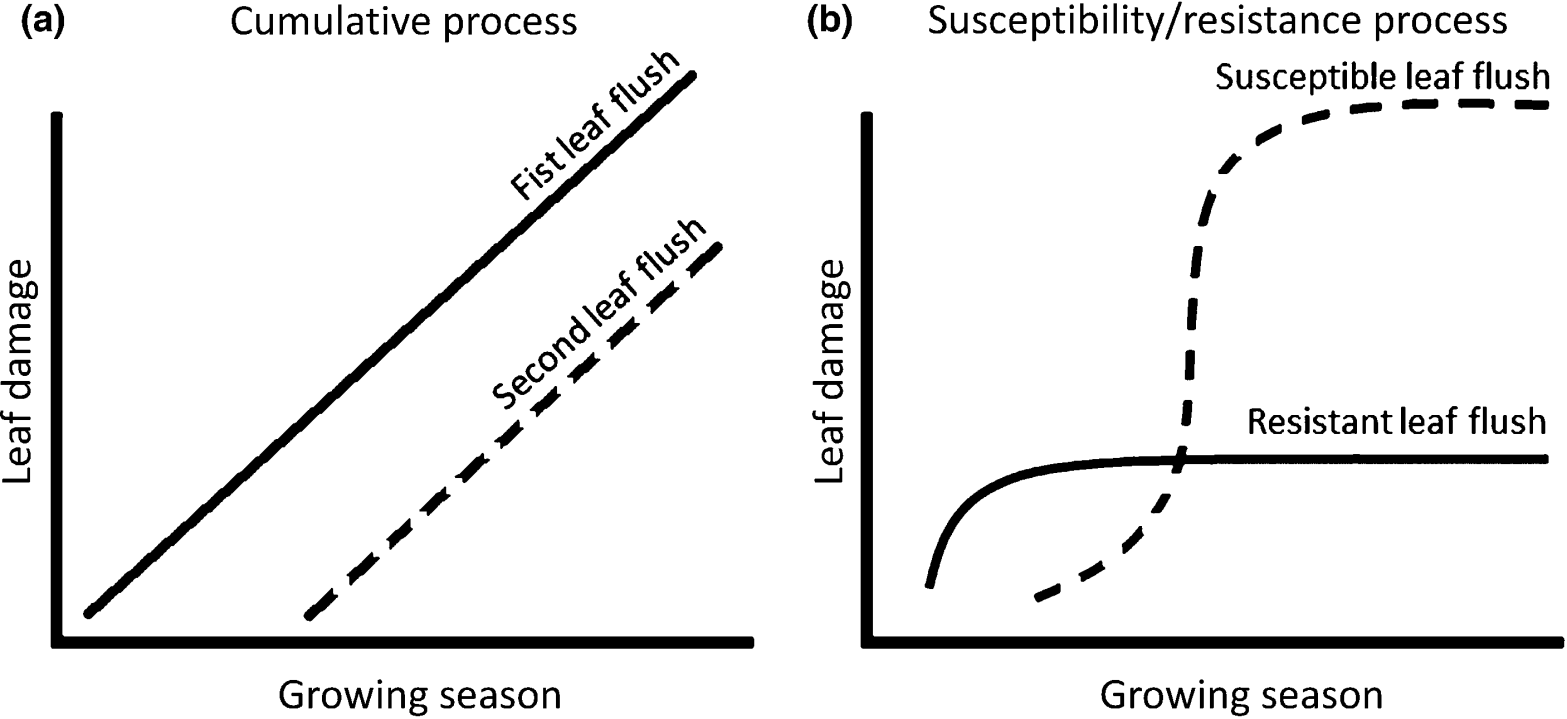
A schematic illustration of two mechanisms that might underlie differences in insect attack and pathogen infection among leaf flushes. (a) The cumulative process, where all leaf flushes accumulate damage through time at an equal rate, and the oldest leaf flushes therefore have a higher level of herbivory and disease at any given time point during the growing season. (b) The susceptibility/resistance mechanism, where – in this case – the first leaf flush is more resistant to attack than the second leaf flush. As a result, the second leaf flush (dashed line) will have higher damage levels than the first leaf flush (continuous line) towards the later part of the growing season.

Insect preference and performance is strongly affected by plant traits such as nutrient content and secondary chemistry, and these same traits are known to affect pathogen performance (Edwards & Ayres, 1982; Auerbach & Simberloff, 1984; Marçais & Desprez‐Loustau, 2014; Jain *et al*., 2019). Yet, we lack insights into how differences in nutrient content and secondary chemistry among different leaf flushes within a single plant individual affect the large within‐plant variation in damage by insects and pathogens that we observe in the field (Gripenberg & Roslin, 2007; Gripenberg *et al*., 2007). We hypothesized that two processes might underlie the effect of leaf flush identity on insect attack and pathogen infection. The first one is a cumulative process, where each leaf flush accumulates damage with the progression of the growing season; hence, at any time during the growing season, the oldest leaf flush is expected to have the highest leaf damage, as followed by the second and subsequent leaf flushes in order of appearance (Fig. 1a). The second one is a resistance/susceptibility process, where the leaf flush with the highest susceptibility during the period of peak insect attack or the highest spore loads has the highest leaf damage (Fig. 1b). Importantly, such processes are nonmutually exclusive. Of the studies to date on differences among leaf flushes in the abundance of insect damage, all four studies on free‐feeding herbivory showed that the first leaf flush has higher levels of free‐feeding herbivory than the second leaf flush (Table 1). In contrast, all three studies on leaf miners reported higher infestation rates on leaves from the second flush (Table 1). To our knowledge, there is no study on galling insects. Finally, the single observational study to date on pathogen infection showed higher infection levels on leaves from the first flush than on those from the second flush (Table 1). Taken together, previous work suggests the highest levels of free‐feeding herbivory and pathogen infection in the first leaf flush, while studies on leaf miners found the highest number of mines in the second leaf flush (Table 1). Yet, no study has directly compared the mechanisms driving differences among leaf flushes among insect guilds, or among members of the insect and microbial kingdom, when sharing the same host plant. Studies so far are also limited in spatial extent, and we lack insights into whether patterns can be generalized across the distribution of the host plant. While rarely tested, differences among the leaf flushes in their primary and secondary chemistry could mediate the relationship between leaf flush identity, insect attack and pathogen infection (Moles & Westoby, 2000; Prado *et al*., 2014; Fuenzalida *et al*., 2019). Yet, the relationship between primary and secondary chemistry and insect and pathogen attack might also differ among leaf flushes.

**Table 1:** Overview of observational studies that report on the relationship between leaf flush identity, insect attack and fungal pathogen infection.

Attacker guild	Study	Plant species	Attacker species	First flush	Second flush	Third flush
Free‐feeders	Fuenzalida *et al*. (2019)	*Nothofagus pumilio*	*Ormiscodes amphimone*	High	Low	Not studied
	Lieberman & Lieberman (1984)	59 species	Free feeders	High	Low	Low
	Moles & Westoby (2000)	51 species	Free feeders	High	Low	Not studied
	Prado *et al*. (2014)	*Zamia stevensonii*	Free feeders	High	Low	High
Leaf miners	Auerbach & Simberloff (1984)	*Quercus nigra*	*Acrocercops* sp. *Neurobathra strigifinitella*	Low	High	Not studied
	Ayabe *et al*. (2015)	*Ligustrum japonicum*	*Phyllocnistis* sp.	Low	High	Not studied
	Potter & Redmond (1989)	*Ilex opaca*	*Phytomyza ilicicola*	Low	High	Not studied
Pathogens	Call & St. Clair (2017)	*Populus tremuloides*	*Drepanopeziza* sp.	High	Low	Not studied

The words ‘High’ and ‘Low’ indicate leaf flushes with high and low levels, respectively, of insect attack and fungal pathogen infection, as compared to the other leaf flushes.

In Lieberman & Lieberman (1984), 179 flushing episodes were recorded in a tropical area of Ghana and the cell for the third leaf flush represents the third and subsequent leaf flushes in this study.

Our overarching aim was to identify the relationships between leaf flush, primary and secondary chemistry of plants, insect attack and pathogen infection during an entire growing season. For this, we surveyed leaves of one of the dominant tree species of temperate deciduous European forests and, i.e. the pedunculate oak (*Quercus robur* L., Fagales: Fagaceae). To achieve generality, we sampled trees in 20 locations across seven countries during the early, mid and late season. More specifically, we targeted the following questions:

1. Do leaf primary (nitrogen and phosphorus) and secondary chemistry (flavonoids, hydroxycinnamic acids, condensed tannins and hydrolysable tannins) differ among leaf flushes during the early, mid and late season?
2. Do insect attack (free‐feeding herbivory, leaf miners and gallers) and pathogen infection differ among leaf flushes during the early, mid and late season?
3. Do primary and secondary chemistry of plants mediate the effect of leaf flush on insect attack and pathogen infection?

## Materials and Methods

### Study species

The pedunculate oak *Q. robur* L. is one of the most dominant tree species in the temperate deciduous forests of Europe and key for wood production. The species grows along a wide range of environmental conditions, including Atlantic, sub‐Mediterranean and oceanic continental climates (Petit *et al*., 2002; Annighöfer *et al*., 2015). The production of multiple leaf flushes is characteristic of oaks (Hilton *et al*., 1987; Moles & Westoby, 2000; Battey, 2003; Elliott *et al*., 2006; Prado *et al*., 2014; Fuenzalida *et al*., 2019), and the production of each leaf flush takes place during a short period of the growing season within a specific area (Hilton *et al*., 1987; Beikircher & Mayr, 2013; Fuenzalida *et al*., 2019). Oaks from the southern range produce three leaf flushes (or more) during the growing season, while a large fraction of oaks in the northern range only has a single leaf flush (Hilton *et al*., 1987). The first oak leaf flush occurs in April in the southern part of the range and in the end of May in the northern part of the range.

Oaks are attacked by a large diversity of insects and pathogens that feed on or infect its leaves (Southwood, 1961; Tack & Roslin, 2011; Marçais & Desprez‐Loustau, 2014). Of the insects, oaks harbour a large community of herbivores including free‐feeders, leaf miners, and gallers (Southwood, 1961; Tack *et al*., 2010; Tack & Roslin, 2011). Of the pathogens, the pedunculate oak is frequently attacked by the oak powdery mildew complex (*Erysiphe* spp., Erysiphales: Erysiphaceae), which consists of the species *Erysiphe alphitoides* Griffon & Maubl., *Erysiphe hypophylla* Nevodosky and *Erysiphe quercicola* Takam. and Braun (Marçais & Desprez‐Loustau, 2014; Desprez‐Loustau *et al*., 2018). *Erysiphe* species grow their mycelium on the upper (adaxial) and/or lower (abaxial) leaf surfaces, and only penetrate the epidermal cells with their feeding organs, which are called haustoria (Bushnell, 1972; Marçais *et al*., 2009; Liu *et al*., 2019). In early spring, the sexual spores (chasmothecia) are released and infect oak leaves. Oak powdery mildew produces asexual spores (conidia) during the rest of the growing season, resulting in multiple asexual generations (Marcais *et al*., 2009; Faticov *et al*., 2020). The pedunculate oak is a suitable model species to answer questions related to the ecological impact of distinct leaf flushes, since it is a widespread foundation tree in Europe that harbours a large diversity of insects and pathogens, and it produces easily distinguishable leaf flushes during distinct time periods.

### Sampling locations and field sampling

**Fig. 2:**
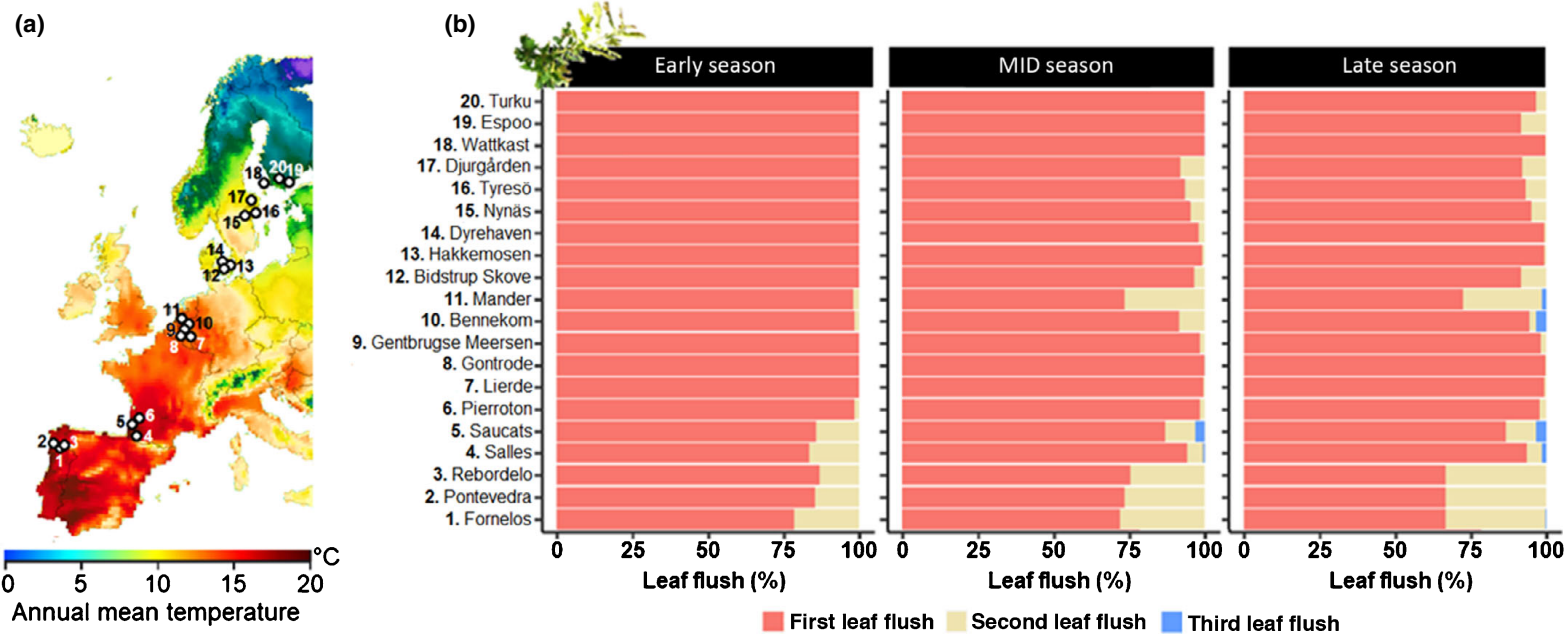
Overview of the location of the study populations and relative abundance of the different oak leaf flushes (*Quercus robur*). (a) Map of Europe with the location of the 20 sampled populations (circles), with the colour gradient indicating annual mean temperature (data source: *Copernicus*). (b) The percentage of shoots that belong to the first, second and third leaf flush, separately for each population during the early, mid and late season.

To assess if primary and secondary plant chemistry, insect attack and pathogen infection differ among leaf flushes across its range, we selected 20 *Q. robur* populations in seven European countries, with a minimum distance of 20 km among populations (Fig. 2). Within each population, we marked three mature oak trees in which we sampled two permanently marked branches during the early (8–12 June), middle (27–31 July) and late growing season (7–18 September). To distinguish leaves belonging to different flushes, shoots were marked using coloured cable ties (Supporting Information Fig. S1). During each season, we visually estimated the percentage of shoots belonging to the first, second and third flush.

To assess primary and secondary plant chemistry, we randomly collected five undamaged leaves (two or three per branch) from each leaf flush (if available), stored them in paper bags and preserved them in cooler boxes during transport. To assess insect attack and pathogen infection, we collected up to 30 randomly‐selected leaves (15 per branch) from each leaf flush (if available) in each tree. All leaves were oven‐dried for 72 h at 40°C. We opted for oven‐drying (rather than e.g. freeze‐drying), as this method was available for all project partners. We sampled leaves of each flush at each sampling date, such that leaves from the first flush were sampled three times at all locations, and, depending on the location, leaves from the second flush were sampled two or three times, and leaves from the third flush were sampled once or twice. We only sampled fully expanded leaves to avoid variation due to rapid changes in leaf chemistry during early leaf development (Salminen *et al*., 2004; Gripenberg *et al*., 2007).

### Quantification of primary and secondary compounds

To quantify leaf nutrient concentrations (nitrogen and phosphorus), we digested *c*. 0.1 g of homogenized dried leaf material (*n* = 270) in a mixture of selenous sulphuric acid and hydrogen peroxide (Moreira *et al*., 2012). Then, we used a colorimetric analysis of diluted aliquots of the digestion to quantify the concentrations (expressed in mg g^−1^ dry tissue) of nitrogen (indophenol blue method) and phosphorus (molybdenum blue method) by using a Bio‐Rad 650 microplate reader (Bio‐Rad Laboratories, Hercules, CA, USA) at 650 nm and 700 nm, respectively (Walinga *et al*., 1995).

To quantify secondary compounds, we extracted phenolics using 20 mg of dry leaf tissue with 1 ml of 70% methanol in an ultrasonic bath for 15 min, followed by centrifugation (Moreira *et al*., 2014). We then transferred these methanolic extracts to chromatographic vials. For phenolic estimation, we used ultrahigh‐performance liquid‐chromatography (UHPLC, Nexera LC‐30AD; Shimadzu, Kyoto, Japan) equipped with a Nexera SIL‐30AC (Shimadzu) injector and a SPD‐M20A ultraviolet‐visible (UV‐vis) photodiode array detector (Shimadzu) connected to quadrupole time‐of‐flight tandem mass spectrometry (UHPLC‐Q‐TOF‐MS/MS; Bruker, Karlsruhe, Germany) with a heated electrospray ionization (ESI) source (Thermo Dionex Ultimate 3000LC; ThermoFisher Scientific, Waltham, MA, USA) (Moreira *et al*., 2020). LC‐Q‐TOF system stability was tested by three consecutive injections of chloramphenicol (ESI negative mode; ΔRT = 0.01 min; Δ*m*/z = 0.002) and triphenyl phosphate (ESI positive mode; ΔRT = 0.02 min; Δ*m*/*z* = 0.001). The compound separation was carried out on a 2.6 µm C18 82–102 Å, LC Column 100 × 4.6 mm (Bruker, Karlsruhe, Germany), protected with a C_18_ guard cartridge (Bruker). The flow rate was 0.4 ml min^−1^ and the oven temperature was set at 25°C. The mobile phase consisted of two solvents: water–formic acid (0.05%) (A) and acetonitrile–formic acid (0.05%) (B), starting with 5% B and using a gradient to obtain 30% B at 4 min, 60% B at 10 min, 80% B at 13 min and 100% B at 15 min. The injection volume was 15 µl. Compound identification was done based on the data from standard substances and published literature, including retention time (tR), maximum wavelength (*λ*_max_), ([M–H]^−^), and major fragment ions. We identified four groups of phenolic compounds: (1) flavonoids; (2) ellagitannins and gallic acid derivatives (‘hydrolysable tannins’ hereafter); (3) proanthocyanidins (‘condensed tannins’ hereafter); and (4) hydroxycinnamic acids. We quantified flavonoids as rutin equivalents, condensed tannins as catechin equivalents, hydrolysable tannins as gallic acid equivalents, and hydroxycinnamic acids as ferulic acid equivalents (Moreira *et al*., 2020). Since the quantification was done by UV detection, gallic acid could be underestimating hydrolysable tannins and catechin the larger condensed tannins, respectively, which may contribute to the relatively low levels of tannins reported (Salminen *et al*., 1999). We achieved the quantification of these phenolic compounds by external calibration using calibration curves at 0.25, 0.5, 1, 2 and 5 μg ml^−1^. We expressed phenolic compound concentrations in mg g^−1^ dry tissue. See the figure and table in Notes S1 for an example of a HPLC chromatogram and a table with compound identity.

### Quantification of insect attack and pathogen infection

To assess damage by insects, we visually estimated the percentage of free‐feeding herbivory and recorded the presence–absence of mines and galls on each leaf (Johnson *et al*., 2016; Barr *et al*., 2021). While *E. alphitoides* and *E. quercicola* are more likely to infect the upper leaf side, *E. hypophylla* only infests the lower leaf side (Desprez‐Loustau *et al*., 2018). Thus, we visually estimated the percentage of the leaf covered by oak powdery mildew, separately for the upper and lower leaf surface (Faticov *et al*., 2021; McClory *et al*., 2021; van Dijk *et al*., 2022). To avoid biases, a single person (AG) measured all 8167 leaves.

### Statistical analyses

To examine differences among leaf flushes in primary and secondary plant chemistry, insect attack and pathogen infection (on both leaf sides), we used generalized linear mixed effects models using the *lmer* and *glmer* functions from the package lme4 in R v.4.0.0 (Bates *et al*., 2015; R Development Core Team, 2020). We assessed model fit using the R‐packages sjPlot and Dharma (Hartig, 2020; Lüdecke, 2020) and tested for statistical significance using the function ANOVA in the R‐package car (Weisberg, 2019). Since not all leaf flushes are present during all seasons, the data collected in this type of study are per definition imbalanced, and we took a two‐step approach to address this problem. First, to account for imbalance in the data during the analysis, we included country, population and tree as nested random factors in the model, and we used type III tests, which are more sensitive to imbalanced data, to test for significance (Bolker *et al*., 2009; Ellison *et al*., 2009). Second, to validate this approach, we also ran models without the third leaf flush, as well as season‐specific and leaf flush‐specific models, which are presented in Tables S3–S22. Importantly, models including or excluding the third leaf flush, and models separately for each season and leaf flush, did not differ qualitatively in their results. We conducted pairwise Tukey tests to test for significant differences between leaf flushes using the R‐package emmeans (Lenth, 2020).

To examine differences among leaf flushes in the concentration of primary compounds (nitrogen and phosphorus), secondary compounds (flavonoids, hydroxycinnamic acids, condensed tannins and hydrolysable tannins), insect attack (percentage of free‐feeding herbivory, proportion of leaves with leaf mines and proportion of leaves with galls) and pathogen infection (separately for the upper and lower leaf surface), we modelled each of these response variables as functions of the categorical fixed effects leaf flush identity (a factor, coded as leaf flush 1, leaf flush 2 and leaf flush 3) and season (a factor, coded as early season, middle season and late season). As the effect of leaf flush might differ among the early, middle and late season, we included the two‐way interaction between leaf flush and season. To account for the hierarchical design, we included country and population as nested within country as random intercepts. To account for repeated measurements, we included tree identity as a random intercept. For free‐feeding herbivory, we specified a Gaussian distribution with an identity link, and for the proportion of leaves with leaf mines and galls, we specified a binomial distribution with a logit link. As leaves from the third leaf flush were rather few, we repeated the same analyses without leaves from the third flush; however, excluding leaves from the third flush did not result in qualitative changes in the results (Tables S1, S2 compared with Tables 2, 3).

**Table 2:** Differences among the three oak leaf flushes (*Quercus robur*) in the concentration of primary compounds (nitrogen and phosphorus) and secondary compounds (flavonoids, hydroxycinnamic acids, condensed tannins and hydrolysable tannins) during the early, mid and late season.

Response variable	Predictor	*χ* ^2^	df	*P*
Nitrogen	Leaf flush	2357.95	2	**<0.001**
	Season	116.63	2	**<0.001**
	Leaf flush × Season	136.21	3	**<0.001**
Phosphorus	Leaf flush	560.56	2	**<0.001**
	Season	897.40	2	**<0.001**
	Leaf flush × Season	420.00	3	**<0.001**
Flavonoids	Leaf flush	1851.89	2	**<0.001**
	Season	1592.06	2	**<0.001**
	Leaf flush × Season	834.17	3	**<0.001**
Hydroxycinnamic acids	Leaf flush	444.37	2	**<0.001**
	Season	521.06	2	**<0.001**
	Leaf flush × Season	78.67	3	**<0.001**
Condensed tannins	Leaf flush	2078.77	2	**<0.001**
	Season	52.80	2	**<0.001**
	Leaf flush × Season	509.83	3	**<0.001**
Hydrolysable tannins	Leaf flush	2738.49	2	**<0.001**
	Season	1037.14	2	**<0.001**
	Leaf flush × Season	1205.87	3	**<0.001**

Shown are *χ* ^2^ values, degrees of freedom (df) and *P*‐values from linear mixed effect models, with significant *P*‐values (*P* < 0.05) in bold.

**Table 3:** Differences among the three oak leaf flushes (*Quercus robur*) in insect attack (percentage of free‐feeding herbivory, proportion of leaves with leaf mines, proportion of leaves with galls) and pathogen infection (percentage of infection by powdery mildew on upper and lower leaf side) during the early, mid and late season.

Response variable	Predictor	*χ* ^2^	df	*P*
Herbivory (%)	Leaf flush	262.15	2	**<0.001**
	Season	37.67	2	**<0.001**
	Leaf flush × Season	5.03	3	0.170
Proportion of leaves with leaf mines	Leaf flush	383.63	2	**<0.001**
	Season	38.61	2	**<0.001**
	Leaf flush × Season	16.33	3	**<0.001**
Proportion of leaves with galls	Leaf flush	131.65	2	**<0.001**
	Season	13.91	2	**0.042**
	Leaf flush × Season	38.09	3	**<0.001**
Infection by powdery mildew on upper leaf side (%)	Leaf flush	2687.52	2	**<0.001**
	Season	398.56	2	**<0.001**
	Leaf flush × Season	86.08	3	**<0.001**
Infection by powdery mildew on lower leaf side (%)	Leaf flush	688.94	2	**<0.001**
	Season	856.21	2	**<0.001**
	Leaf flush × Season	422.63	3	**<0.001**

Shown are *χ* ^2^ values, degrees of freedom (df) and *P*‐values from generalized linear mixed effect models, with significant *P*‐values (*P* < 0.05) in bold.

We next investigated to what extent plant chemistry mediates the effect of leaf flush identity on insect attack and pathogen infection. As we detected large variation in the relationships between plant chemistry and attackers between leaf flushes and seasons (Figs S1–S16), we opted for two complementary sets of models, i.e. season‐specific and leaf flush‐specific generalized linear mixed effects models. In the season‐specific models, we explored the relationships among primary and secondary plant compounds, insect attack and pathogen infection separately for each season, where we fitted the response variables (percentage of free‐feeding herbivory, proportion of leaves with mines, proportion of leaves with galls, area covered by powdery mildew on the upper leaf side and area covered by powdery mildew on lower leaf side) as functions of the predictors leaf flush, primary (nitrogen and phosphorus) and secondary compounds (flavonoids, hydroxycinnamic acids, condensed tannins, hydrolysable tannins). As the relationships among plant chemistry, insect attack and pathogen infection might differ among leaf flushes, we included all two‐way interactions among leaf flush and chemical compounds. In the leaf flush‐specific models, we explored the relationships between primary and secondary plant compounds and insect attack and pathogen infection separately for each flush, where we fitted the response variables as functions of the predictors season and primary and secondary compounds. As the relationships between plant chemistry, insect attack and pathogen infection might differ among the seasons, we included all two‐way interactions between season and chemical compounds. To account for the hierarchical and repeated‐measures design, we included country, population and tree identity as random intercepts in all these models.

## Results

The relative abundance of leaves from the second and third flushes was highly variable among countries, and showed a tendency to increase from north to south and with the progression of the growing season (Fig. 2b; Table S23). Towards the end of the growing season, the majority (89.9%) of leaves belonged to the first flush, 9.6% to the second flush, and only 0.5% to the third flush.

### Differences among leaf flushes in primary and secondary chemistry

**Fig. 3:**
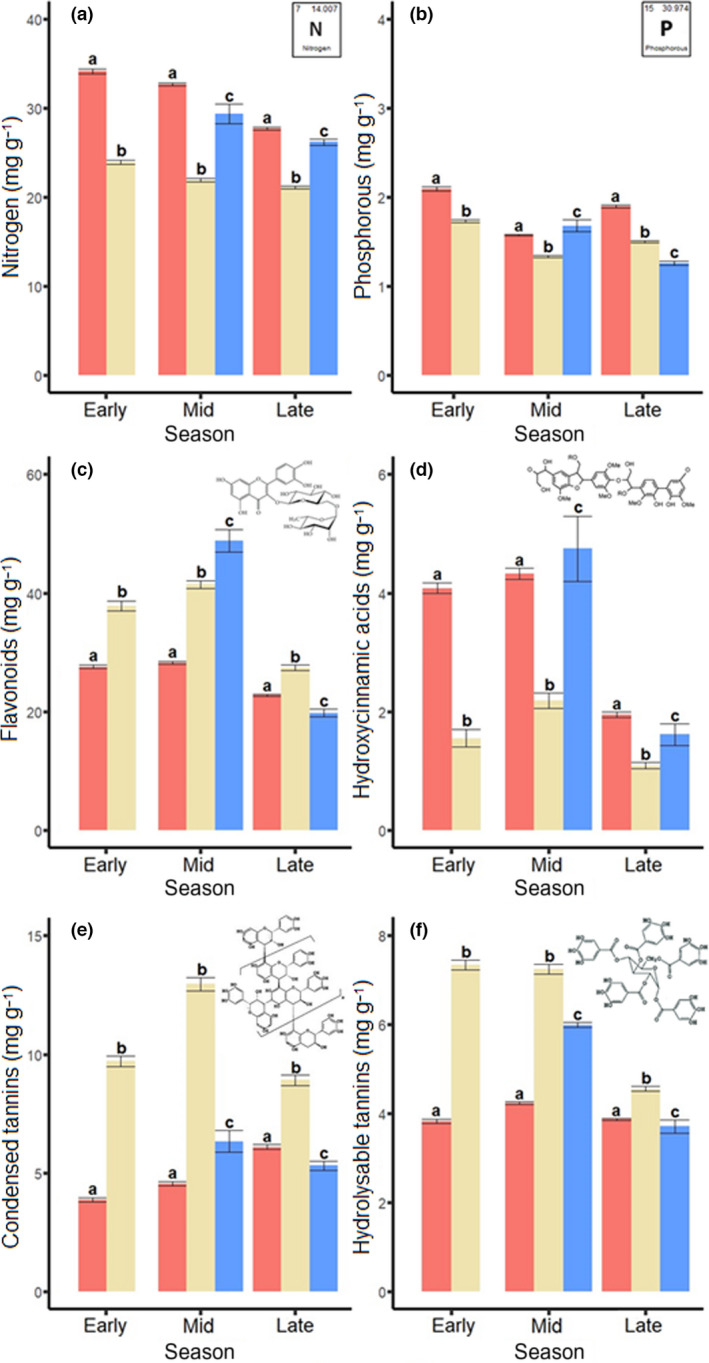
Differences among oak leaf flushes (*Quercus robur*) in the concentration of primary compounds (a) nitrogen and (b) phosphorus and secondary compounds (c) flavonoids, (d) hydroxycinnamic acids, (e) condensed tannins and (f) hydrolysable tannins during the early, mid and late season. Concentrations are expressed in mg g^−1^ of dry tissue. Bars represent means and error bars represent standard errors. Significant differences among leaf flushes in each season are indicated by different letters above bars (*P* < 0.05), with statistical details provided in Supporting Information Table S25.

Differences in the concentration of primary and secondary compounds among leaves from the first, second and third flushes were inconsistent among the early, mid and late season, as indicated by the significant interaction terms (Fig. 3; Table 2).

For nitrogen, the interactive effect was relatively weak (Fig. 3a), and the concentration of nitrogen was highest in leaves from the first flush, intermediate in leaves from the third flush, and lowest in leaves from the second flush during all parts of the growing season (Fig. 3a; Table 2). In each of the leaf flushes there was a decrease in nitrogen concentration with the progression of the growing season (Figs 3a, S17; Tables 2, S24). For phosphorus, the concentration was consistently lower in leaves from the second flush than in leaves from the first flush, but leaves from the third flush changed rank during the growing season (Fig. 3b; Table 2). Unlike for nitrogen, there was no clear change in phosphorus concentration with the progression of the growing season (Figs 3b, S17).

The concentration of flavonoids, condensed tannins and hydrolysable tannins were consistently higher, and the concentration of hydroxycinnamic acids was consistently lower, in leaves from the second flush as compared to leaves from the first flush, although the effect size differed strongly among the seasons (Figs 3c–f, S17; Tables 2, S24). In contrast to the consistent difference in the concentration of secondary compounds between leaves from the first and second flushes, leaves from the third flush changed rank during the growing season (Figs 3c–f, S17; Tables 2, S24).

### Differences among leaf flushes in insect attack and pathogen infection

**Fig. 4:**
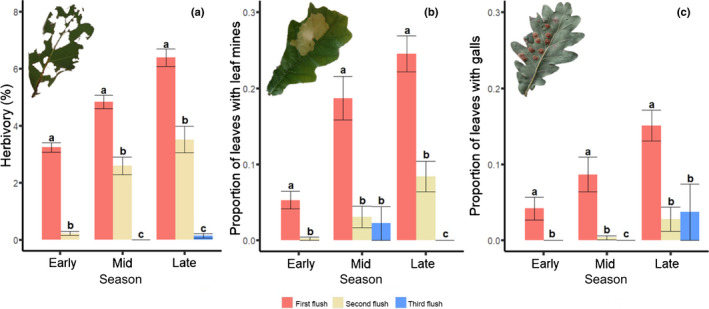
Differences among oak leaf flushes (*Quercus robur*) in insect attack during the early, mid and late season: (a) percentage of herbivory, (b) proportion of leaves with leaf mines and (c) proportion of leaves with galls. Bars represent means and error bars represent standard errors. Significant differences among leaf flushes in each season are indicated by letters above bars (*P* < 0.05), with statistical details provided in Supporting Information Table S25.

For all three herbivore guilds, the amount of damage increased strongly during the growing season (Fig. 4; Table 3). The percentage of free‐feeding herbivory was consistently highest in leaves from the first flush, intermediate in leaves from the second flush and lowest in leaves from the third flush (Fig. 4a; Table 3). The proportions of leaves with mines and galls were several‐fold higher in leaves from the first flush than in leaves from the second flush, whereas the number of leaves from the third flush were too few to obtain accurate estimates given the low infestation levels (Fig. 4b,c).

**Fig. 5:**
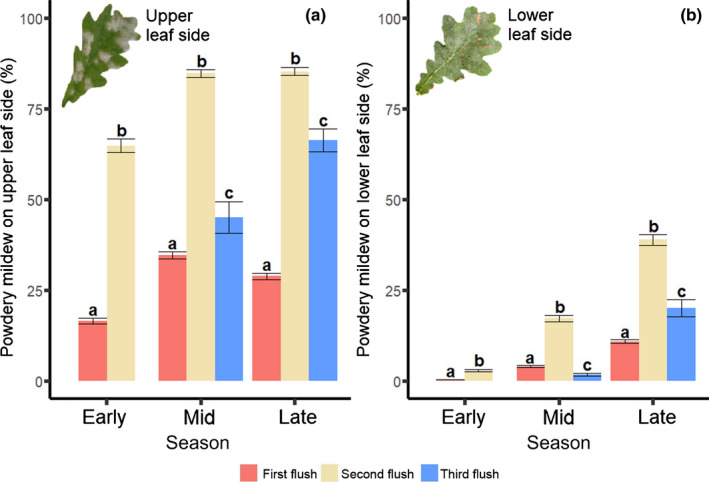
Differences among oak leaf flushes (*Quercus robur*) in infection by oak powdery mildew (*Erysiphe* spp.) on the (a) upper and (b) lower leaf side of the pedunculate oak *Quercus robur* during the early, mid and late season. Bars represent means and error bars represent standard errors. Significant differences among leaf flushes in each season are indicated by letters above bars (*P* < 0.05), with statistical details provided in Supporting Information Table S25.

Overall, oak powdery mildew infection levels were higher on the upper than on the lower side of the leaf (Fig. 5). On both leaf sides, infection was higher in leaves from the second flush than in leaves from the first flush during all parts of the growing season, whereas leaves from the third flush tended to have intermediate infection levels, even though the effect sizes differed among the early, mid and late season (Fig. 5; Table 3).

### The link between leaf flush identity, primary and secondary chemistry, and damage by insects and pathogens

The relationship between each primary (nitrogen and phosphorus) and secondary compound (flavonoids, hydroxycinnamic acids, condensed tannins and hydrolysable tannins) and insect attack (free‐feeding herbivory, leaf miners and gallers) or pathogen infection (on upper and lower side) differed strongly among leaf flushes (Tables S3–S12) and among seasons (Tables S13–S22). Thus, it is hard to highlight any general pattern on the relationship between plant chemistry and insect attack and pathogen infection (Figs S1–S16). As an example of the inconsistency of the relationship between leaf chemistry and damage among seasons, the levels of infection by oak powdery mildew on the upper leaf side was lower in leaves that had the highest concentrations of flavonoids during the early and late season, but not during the mid season (Figs S12–S14; Table S20). As an example of the inconsistency of the relationship between leaf chemistry and damage among leaf flushes, we detected no relationship between free‐feeding herbivory and the concentrations of flavonoids, hydroxycinnamic acids and hydrolysable tannins in leaves from the first flush, while free‐feeding herbivory rates were negatively related with flavonoids, hydroxycinnamic acids and hydrolysable tannins in leaves from the second leaf flush (Figs S2–S4; Table S4).

## Discussion

To disentangle the effects of leaf flush identity on within‐plant variation in primary and secondary chemistry, herbivory and pathogen infection, we sampled the different leaf flushes in 20 oak populations in seven European countries during the early, middle and late season. In general, leaves from the first flush had higher levels of nitrogen, phosphorus and hydroxycinnamic acids, whereas they had lower levels of flavonoids, condensed tannins and hydrolysable tannins, compared to leaves from the second flush. Plant chemistry was highly variable for leaves from the third flush. Damage by free‐feeding herbivores, leaf miners and gallers matched the prediction that insect herbivory increases cumulatively during the growing season, with infestation levels increasing with the progression of the growing season and decreasing from the first to the third leaf flush. In contrast, infection by oak powdery mildew was highest on the second leaf flush, suggesting that the process of resistance/susceptibility plays a major role in understanding pathogen infection levels. The relationship between plant chemistry, insect attack and pathogen infection differed among flushes and seasons, precluding any general patterns. Our findings demonstrate that leaf flush identity can explain a large amount of within‐plant variation in chemistry, insect attack and pathogen infection, and should thus be taken into account in future studies on plant chemistry and species interactions. As climate change is expected to increase the frequency of the second and subsequent leaf flushes, we predict accompanying changes in the patterns of leaf chemistry, insect attack and pathogen infection.

Leaves from the first flush generally had higher concentrations of nitrogen, phosphorus and hydroxycinnamic acids, whereas they had lower concentrations of flavonoids, condensed tannins and hydrolysable tannins. Our results match with previous studies, which consistently found that leaves from the second flush have lower nutrient contents (e.g. Bryant *et al*., 1983; Fuenzalida *et al*., 2019) and higher concentrations of secondary compounds (e.g. Potter & Redmond, 1989; Krause *et al*., 1993; St. Clair *et al*., 2009). For example, Fuenzalida *et al*. (2019) found that the first leaf flush of *Nothofagus pumilio* had higher nutrient concentrations than the second leaf flush, while Potter & Redmond (1989) reported that the second leaf flush of *Ilex opaca* had higher levels of chemical defences against herbivory than the first leaf flush. This raises an interesting evolutionary question: Why are leaves that are present for the entire growing season, and will face higher levels of insect damage, also those that are least defended? One possible reason might be a trade‐off between rapid growth of large numbers of leaves in spring and the production of secondary compounds, for example due to limited resources at the start of the growing season (the growth‐differentiation balance hypothesis; Riipi *et al*., 2002; Glynn *et al*., 2007; Elger *et al*., 2009). While our study focused on leaf chemistry, future studies might also explore the role of physical defences, such as leaf toughness and trichomes, in shaping differences in insect attack and pathogen infection among leaf flushes and seasons.

We found that damage by free‐feeders, leaf miners and gallers decreased from leaves from the first flush to leaves from the third flush, indicating that herbivory follows a cumulative process (Fig. 1a). The observation of the highest levels of free‐feeding herbivory on leaves from the first leaf flush matches with the four previous studies (Table 1). For example, Lieberman & Lieberman (1984) found that herbivore damage was higher on the first leaf flush than on subsequent leaf flushes for 59 plant species from dry tropical forests in Ghana, and Moles & Westoby (2000) found that free‐feeding herbivory was higher in leaves from the first flush of 51 plant species from the area of Sydney, Australia. Our findings of a higher incidence of leaf miners on older leaf flushes strikingly contrasts with the three previous studies on the effect of leaf flush identity on leaf miners, which all observed a higher number of mines on leaves from the second flush (Auerbach & Simberloff, 1984; Potter & Redmond, 1989; Ayabe *et al*., 2015). For example, Auerbach & Simberloff (1984) found a higher number of mines of *Acrocercops* sp. (Lepidoptera: Gracillariidae) and *Neurobathra strigifinitella* (Lepidoptera: Gracillariidae) on leaves from the second flush of *Quercus nigra* (Fagales: Fagaceae), and Ayabe *et al*. (2015) demonstrated that *Phyllocnistis* sp. (Lepidoptera: Gracillariidae) had a preference for the young leaves from the second flush of *Ligustrum japonicum* (Lamiales: Oleaceae). While speculative, these contrasting findings might be explained by the fact that previous studies focused on single leaf miner species that are unrepresentative of the full leaf miner community, or that patterns are dependent on the identity of the plant or insect species. Since there are no comparable studies for gallers, additional studies are needed to assess the generality of our results. Taken together, the evidence suggests that free‐feeding herbivory is generally a cumulative process (Fig. 1a), while damage by leaf miners can – depending on the plant or insect species – be a consequence of cumulative damage and/or susceptibility (Fig. 1). The question of generality is still out for galling insects.

In contrast to insect attacks, we found that pathogen infection was lowest on leaves from the first flush, highest on leaves from the second flush, and intermediate on leaves from the third flush. This indicates a role for the process of susceptibility/resistance, and suggests that pathogen infection is not (purely) based on the accumulation of infection through time. In the case of powdery mildew on the pedunculate oak, where developing leaves are known to be more susceptible than mature leaves (Edwards & Ayres, 1982; Marcais *et al*., 2009), we propose the following scenario. In early spring, fungal spore load in the air is relatively low, and many developing leaves from the first flush escape infection. During the late spring and summer season, the oak powdery mildew spore load will rapidly increase, and this time period matches with the time of development of leaves from the second flush, which will develop high levels of pathogen infection (Marçais *et al*., 2009; Desprez‐Loustau *et al*., 2010). During the late summer and early autumn, oak powdery mildew spore production might decrease due to less favourable environmental conditions (Marçais & Desprez‐Loustau, 2014), leading to intermediate infection levels on leaves from the third flush. Importantly, the processes of accumulation and susceptibility/resistance are nonmutually exclusive, and the higher levels of pathogen infection on leaves from the second than the third flush might also be (partly) explained by the process of accumulation of damage through time. The finding of the lowest infection levels on leaves from the first flush contrasts with the pattern found in the only other available study on the relationship among leaf flush identity and pathogen infection levels: Call & St. Clair (2017) found the highest levels of infection by *Drepanopeziza* sp. (Helotiales: Drepanopezizaceae) in leaves from the first flush of *Populus tremuloides* (Malpighiales: Salicaceae). Based on our findings and the literature, we therefore conclude that the impact of leaf flush identity on pathogen infection is dependent on the identity of the pathogen and/or plant species, which makes sense, as the process of susceptibility/resistance is well‐known to depend on the specific combination of plant and pathogen species (Jain *et al*., 2019). Importantly, for the combination of oak and oak powdery mildew, it seems that pathogen infection is not (only) based on accumulation through time, but rather driven by the high susceptibility of developing leaf flushes, resulting in high levels of infection when high levels of infectious spores are present in the environment.

Our findings showed that the relationships between plant chemistry, insect attack and pathogen infection were highly variable among leaf flushes and seasons, precluding the identification of a few simple and general patterns. Yet, if the variability in the relationships between plant chemistry, insect attack and pathogen infection among leaf flushes is a general pattern across the plant kingdom, it has major methodological implications: future studies examining these relationships will have to take into account leaf flush identity when sampling leaves in the field or within the confines of experiments. Regarding the seasonal variation in the relationships detected, one promising direction might be to use a sampling design with very high temporal resolution to establish the seasonal trajectory of the relationships among plant chemistry, insect attack and pathogen infection. We propose two further research directions based on our general findings. First, future studies might quantify the relative importance of leaf flush identity in shaping intra‐specific variation in leaf chemistry, insect attack and pathogen infection across hierarchical spatial scales (i.e. leaf, shoot, branch and tree) at different parts of the growing season, which would require sampling leaves belonging to the different leaf flushes proportionally to their relative abundance on the tree (i.e. randomly sample across all leaf flushes). Second, studies might explore the existence of geographic variation in the effect of leaf flush identity on leaf chemistry, insect attack and pathogen infection, and if such variation exists, identify the underlying environmental drivers. From a methodological perspective, we note that seasonal studies on the effect of leaf flush will by definition face imbalanced, and careful validation of robust statistical analyses, for example by the exploration of subsets of the data and/or season‐specific or leaf flush‐specific models, is required.

### Conclusions

Our results highlight that the co‐existence of leaves from different flushes is a major source of within‐plant variation in chemistry, insect attack and pathogen infection. For a dominant tree species in Europe, this pattern is driven by multiple processes: insect attack is higher in earlier leaf flushes via a cumulative process, whereas pathogen infection is higher in later leaf flushes via the process of resistance/susceptibility. We hope that future studies will assess the generality of the importance of leaf flush identity for within‐plant variation, the generality of the relative importance of the different processes driving insect attack and pathogen infection, as well as the functional consequences in terms of plant growth and fitness. As climate change is expected to alter the frequency of second and subsequent leaf flushes, this may in turn lead to changes in the patterns of insect attack and disease levels. A more thorough understanding of the ecological and evolutionary consequences of leaf flush dynamics for patterns of species interactions will thus be important for our ability to predict how large trees and their associated communities of herbivores and pathogens will be affected by global warming.

## Competing interests

All authors declare that there is no conflict of interest.

## Author contributions

AG, KG and AJMT planned and designed the research. AG, XM, BC, IVH, PDF, CM, BGHT, JPJGTH, PUR, NB, RJ, PP and SS conducted fieldwork. XM and FC conducted laboratory work. AG and AJMT analysed data and wrote the manuscript. All authors reviewed the latest version of the manuscript.

## Supporting information


**Fig. S1** An example to illustrate how we distinguished leaves from different leaf flushes, and to clarify our sampling strategy where we randomly sampled leaves within the subset of leaves belonging to a given flush.
**Fig. S2** The effect of concentrations of nitrogen, phosphorus, flavonoids, hydroxycinnamic acids, condensed tannins and hydrolysable tannins on the percentage of free‐feeding herbivory during the early season.
**Fig. S3** The effect of concentrations of nitrogen, phosphorus, flavonoids, hydroxycinnamic acids, condensed tannins and hydrolysable tannins on the percentage of free‐feeding herbivory during the mid season.
**Fig. S4** The effect of concentrations of nitrogen, phosphorus, flavonoids, hydroxycinnamic acids, condensed tannins and hydrolysable tannins on the percentage of free‐feeding herbivory during the late season.
**Fig. S5** The effect of concentrations of nitrogen, phosphorus, flavonoids, hydroxycinnamic acids, condensed tannins and hydrolysable tannins on the proportion of leaves with leaf mines during the early season.
**Fig. S6** The effect of concentrations of nitrogen, phosphorus, flavonoids, hydroxycinnamic acids, condensed tannins and hydrolysable tannins on the proportion of leaves with leaf mines during the mid season.
**Fig. S7** The effect of concentrations of nitrogen, phosphorus, flavonoids, hydroxycinnamic acids, condensed tannins and hydrolysable tannins on the proportion of leaves with leaf mines during the late season.
**Fig. S8**. The effect of concentrations of nitrogen, phosphorus, flavonoids, hydroxycinnamic acids, condensed tannins and hydrolysable tannins on the proportion of leaves with galls during the early season.
**Fig. S9** The effect of concentrations of nitrogen, phosphorus, flavonoids, hydroxycinnamic acids, condensed tannins and hydrolysable tannins on the proportion of leaves with galls during the mid season.
**Fig. S10** The effect of concentrations of nitrogen, phosphorus, flavonoids, hydroxycinnamic acids, condensed tannins and hydrolysable tannins on the proportion of leaves with galls during the late season
**Fig. S11** The effect of concentrations of nitrogen, phosphorus, flavonoids, hydroxycinnamic acids, condensed tannins and hydrolysable tannins in infection by oak powdery mildew (*Erysiphe* spp.) on the upper leaf side of the pedunculate oak *Quercus robur* during the early season.
**Fig. S12** The effect of concentrations of nitrogen, phosphorus, flavonoids, hydroxycinnamic acids, condensed tannins and hydrolysable tannins in infection by oak powdery mildew (*Erysiphe* spp.) on the upper leaf side of the pedunculate oak *Quercus robur* during the mid season.
**Fig. S13** The effect of concentrations of nitrogen, phosphorus, flavonoids, hydroxycinnamic acids, condensed tannins and hydrolysable tannins in infection by oak powdery mildew (*Erysiphe* spp.) on the upper leaf side of the pedunculate oak *Quercus robur* during the late season.
**Fig. S14** The effect of concentrations of nitrogen, phosphorus, flavonoids, hydroxycinnamic acids, condensed tannins and hydrolysable tannins in infection by oak powdery mildew (*Erysiphe* spp.) on the lower leaf side of the pedunculate oak *Quercus robur* during the early season.
**Fig. S15** The effect of concentrations of nitrogen, phosphorus, flavonoids, hydroxycinnamic acids, condensed tannins and hydrolysable tannins in infection by oak powdery mildew (*Erysiphe* spp.) on the lower leaf side of the pedunculate oak *Quercus robur* during the mid season.
**Fig. S16** The effect of concentrations of nitrogen, phosphorus, flavonoids, hydroxycinnamic acids, condensed tannins and hydrolysable tannins in infection by oak powdery mildew (*Erysiphe* spp.) on the lower leaf side of the pedunculate oak *Quercus robur* during the late season.
**Fig. S17** Differences among leaf flushes in the concentration of primary and secondary compounds during the early, mid and late season.
**Notes S1** Example of high‐performance liquid chromatography (HPLC) chromatogram with all the compounds detected.
**Table S1** Differences between the first and the second leaf flush in the concentration of primary and secondary compounds during the early, mid and late season.
**Table S2** Differences between the first and the second leaf flush in insect attack and pathogen infection the early, mid and late season.
**Table S3** The effect of season, nitrogen, phosphorus and their interactions on the percentage of herbivory, separately for the first and second leaf flush.
**Table S4** The effect of season, flavonoids, hydroxycinnamic acids, condensed tannins, hydrolysable tannins and their interactions on the percentage of herbivory, separately for the first and second leaf flush.
**Table S5** The effect of season, nitrogen, phosphorus and their interactions on the proportion of leaves with leaf mines separately for the first and the second leaf flush.
**Table S6** The effect of season, flavonoids, hydroxycinnamic acids, condensed tannins, hydrolysable tannins and their interactions on the proportion of leaves separately for the first and the second leaf flush.
**Table S7** The effect of season, nitrogen, phosphorus and their interactions on the proportion of leaves with galls separately for the first and the second leaf flush.
**Table S8** The effect of season, flavonoids, hydroxycinnamic acids, condensed tannins, hydrolysable tannins and their interactions on the proportion of leaves separately for the first and the second leaf flush.
**Table S9** The effect of seasons, nitrogen, phosphorus and their interactions on the percentage of infection by oak powdery mildew on the upper leaf side separately for the first and the second leaf flush.
**Table S10** The effect of season, flavonoids, hydroxycinnamic acids, condensed tannins, hydrolysable tannins and their interactions on the percentage of infection by oak powdery mildew on the upper leaf side separately for the first and the second leaf flush.
**Table S11** The effect of seasons, nitrogen, phosphorus and their interactions on the percentage of infection by oak powdery mildew on the lower leaf side separately for the first and the second leaf flush.
**Table S12** The effect of seasons, flavonoids, hydroxycinnamic acids, condensed tannins, hydrolysable tannins and their interactions on the percentage of infection by oak powdery mildew on the lower leaf side separately for the first and the second leaf flush.
**Table S13** The effect of nitrogen, phosphorus and their interactions on the percentage of herbivory separately for the early, mid and late season.
**Table S14** The effect of leaf flush, flavonoids, hydroxycinnamic acids, condensed tannins, hydrolysable tannins and their interactions on the percentage of herbivory separately for the early, mid and late season.
**Table S15** The effect of nitrogen, phosphorus and their interactions on the proportion of leaves with leaf mines separately for the early, mid and late season.
**Table S16** The effect of leaf flush, flavonoids, hydroxycinnamic acids, condensed tannins, hydrolysable tannins and their interactions on the proportion of leaves with leaf mines separately for the early, mid and late season.
**Table S17** The effect of nitrogen, phosphorus and their interactions on the proportion of leaves with galls separately for the early, mid and late season.
**Table S18** The effect of leaf flush, flavonoids, hydroxycinnamic acids, condensed tannins, hydrolysable tannins and their interactions on the proportion of leaves with galls separately for the early, mid and late season.
**Table S19** The effect of nitrogen, phosphorus and their interactions on the percentage of infection by oak powdery mildew on the upper leaf separately for the early, mid and late season.
**Table S20** The effect of leaf flush, flavonoids, hydroxycinnamic acids, condensed tannins, hydrolysable tannins and their interactions on the percentage of infection by oak powdery mildew on the upper leaf side separately for the early, mid and late season.
**Table S21** The effect of nitrogen, phosphorus and their interactions on the percentage of infection by oak powdery mildew on the lower leaf side separately for the early, mid and late season.
**Table S22** The effect of leaf flush, flavonoids, hydroxycinnamic acids, condensed tannins, hydrolysable tannins and their interactions on the percentage of infection by oak powdery mildew on the lower leaf side separately for the early, mid and late season.
**Table S23** The impact of latitude on the relative abundance of the first, second and third leaf flush. To account for the hierarchical and repeated‐measures design, we included country, population and tree identity as random intercepts.
**Table S24** Results of paired tests comparing the concentration of primary, secondary compounds, insect attack and pathogen infection among seasons.
**Table S25** Results of paired tests comparing the concentration of primary, secondary compounds, insect attack and pathogen infection among leaf flushes during the early, mid and late seasons.
Please note: Wiley Blackwell are not responsible for the content or functionality of any Supporting Information supplied by the authors. Any queries (other than missing material) should be directed to the *New Phytologist* Central Office.

## Data Availability

The data that support the findings of this study are available on request from the corresponding author.
