# Supplementary material for: The co‐existence of multiple oak leaf flushes contributes to the large within‐tree variation in chemistry, insect attack and pathogen infection
Source: New Phytol. 2022 May 26;235(4):1615–28. doi: 10.1111/nph.18209 (PMC9545873; doi:10.1111/nph.18209)
Supplement: Supplementary file 1 — Fig. S1 An example to illustrate how we distinguished leaves from different leaf flushes, and to clarify our sampling strategy where we randomly sampled leaves within the subset of leaves belonging to a given flush. Fig. S2 The effect of concentrations of nitrogen, phosphorus, flavonoids, hydroxycinnamic acids, condensed tannins and hydrolysable tannins on the percentage of free‐feeding herbivory during the early season. Fig. S3 The effect of concentrations of nitrogen, phosphorus, flavonoids, hydroxycinnamic acids, condensed tannins and hydrolysable tannins on the percentage of free‐feeding herbivory during the mid season. Fig. S4 The effect of concentrations of nitrogen, phosphorus, flavonoids, hydroxycinnamic acids, condensed tannins and hydrolysable tannins on the percentage of free‐feeding herbivory during the late season. Fig. S5 The effect of concentrations of nitrogen, phosphorus, flavonoids, hydroxycinnamic acids, condensed tannins and hydrolysable tannins on the proportion of leaves with leaf mines during the early season. Fig. S6 The effect of concentrations of nitrogen, phosphorus, flavonoids, hydroxycinnamic acids, condensed tannins and hydrolysable tannins on the proportion of leaves with leaf mines during the mid season. Fig. S7 The effect of concentrations of nitrogen, phosphorus, flavonoids, hydroxycinnamic acids, condensed tannins and hydrolysable tannins on the proportion of leaves with leaf mines during the late season. Fig. S8. The effect of concentrations of nitrogen, phosphorus, flavonoids, hydroxycinnamic acids, condensed tannins and hydrolysable tannins on the proportion of leaves with galls during the early season. Fig. S9 The effect of concentrations of nitrogen, phosphorus, flavonoids, hydroxycinnamic acids, condensed tannins and hydrolysable tannins on the proportion of leaves with galls during the mid season. Fig. S10 The effect of concentrations of nitrogen, phosphorus, flavonoids, hydroxycinnamic acids, condensed tannins and hydrolysa [file NPH-235-1615-s001.pdf]

The co-existence of multiple oak leaf flushes contributes to the large within-tree variation in chemistry, insect attack and pathogen infection

Álvaro Gaytán, Xoaquín Moreira, Bastien Castagneyrol, Inge Van Halder, Pieter De Frenne, Camille Meeussen, Bart G.H. Timmermans, Jan P.J.G. Ten Hoopen, Pil U. Rasmussen, Nick Bos, Raimo Jaatinen, Pertti Pulkkinen, Sara Söderlund, Felisa Covelo, Karl Gotthard and Ayco J.M. Tack

Acceptance date: 3 May 2022

SUPPORTING INFORMATION

Table S1. Differences between the first and the second oak leaf flush (*Quercus robur*) in the concentration of primary (nitrogen, phosphorus) and secondary compounds (flavonoids, hydroxycinnamic acids, condensed tannins and hydrolysable tannins) during the early, mid and late season. Shown are χ^2 values, degrees of freedom and p-values from linear mixed effect models, with significant p-values ($p < 0.05$) in bold.

**Response variable**	**Predictor**	χ^2	**df**	**p**
Nitrogen	Leaf flush	2454.81	1	**<0.01**
	Season	1128.95	2	**<0.01**
	Leaf flush × Season	131.61	2	**<0.01**
Phosphorus	Leaf flush	306.02	1	**<0.01**
	Season	2331.29	2	**<0.01**
	Leaf flush × Season	210.46	2	**<0.01**
Flavonoids	Leaf flush	1616.96	1	**<0.01**
	Season	1615.63	2	**<0.01**
	Leaf flush × Season	653.78	2	**<0.01**
Hydroxycinnamic acids	Leaf flush	392.40	1	**<0.01**
	Season	1165.19	2	**<0.01**
	Leaf flush × Season	34.54	2	**<0.01**
Condensed tannins	Leaf flush	2239.21	1	**<0.01**
	Season	170.10	2	**<0.01**
	Leaf flush × Season	506.86	2	**<0.01**
Hydrolysable tannins	Leaf flush	2411.10	1	**<0.01**
	Season	793.58	2	**<0.01**
	Leaf flush × Season	1136.94	2	**<0.01**

Table S2. Differences between the first and the second oak leaf flush (*Quercus robur*) in insect attack (percentage of free-feeding herbivory, proportion of leaves with leaf mines, proportion of leaves with galls) and pathogen infection (percentage of infection by powdery mildew on upper and lower leaf side) during the early, mid and late season. Shown are χ^2 values, degrees of freedom and p-values from linear mixed effect models, with significant p-values ($p < 0.05$) in bold.

**Response variable**	**Predictor**	χ^2	**df**	**p**
Herbivory (%)	Leaf flush	227.57	1	**<0.01**
	Season	130.39	2	**<0.01**
	Leaf flush × Season	4.17	2	0.12
Proportion of leaves with leaf mines	Leaf flush	73.71	1	**<0.01**
	Season	67.61	2	**<0.01**
	Leaf flush × Season	1.87	2	0.39
Proportion of leaves with galls	Leaf flush	24.48	1	**<0.01**
	Season	21.42	2	**<0.01**
	Leaf flush × Season	5.13	2	0.08
Infection by powdery mildew on upper leaf side (%)	Leaf flush	3453.22	1	**<0.01**
	Season	767.45	2	**<0.01**
	Leaf flush × Season	95.61	2	**<0.01**
Infection by powdery mildew on lower leaf side (%)	Leaf flush	809.22	1	**<0.01**
	Season	1006.55	2	**<0.01**
	Leaf flush × Season	418.15	2	**<0.01**

Table S3. The effect of season, nitrogen, phosphorus and their interactions on the percentage of herbivory, separately for the first and second oak leaf flush (*Quercus robur*). Shown are χ^2 values, degrees of freedom and p-values from linear mixed effect models, with significant p-values ($p < 0.05$) in bold.

**Response variable**	**Predictor**	χ^2	**df**	**p**
Herbivory (%) First flush	Season	125.97	2	**<0.01**
	Nitrogen	24.46	1	**<0.01**
	Phosphorus	0.07	1	0.80
	Season × Nitrogen	23.66	2	**<0.01**
	Season × Phosphorus	40.89	2	**<0.01**
Herbivory (%) Second flush	Season	86.18	2	**<0.01**
	Nitrogen	9.91	1	**<0.01**
	Phosphorus	10.27	1	**<0.01**
	Season × Nitrogen	186.69	2	**<0.01**
	Season × Phosphorus	132.43	2	**<0.01**

Table S4. The effect of season, flavonoids, hydroxycinnamic acids, condensed tannins, hydrolysable tannins and their interactions on the percentage of herbivory, separately for the first and second oak leaf flush (*Quercus robur*). Shown are χ^2 values, degrees of freedom and p-values from linear mixed effect models, with significant p-values ($p < 0.05$) in bold.

**Response variable**	**Predictor**	χ^2	**df**	**p**
Herbivory (%) First flush	Season	107.37	2	**<0.01**
	Flavonoids	0.12	1	0.72
	Hydroxycinnamic acids	0.16	1	0.69
	Condensed tannins	4.51	1	**0.03**
	Hydrolysable tannins	1.07	1	0.30
	Season × Flavonoids	15.80	2	**<0.01**
	Season × Hydroxycinnamic acids	13.21	2	**<0.01**
	Season × Condensed tannins	38.91	2	**<0.01**
	Season × Hydrolysable tannins	17.09	2	**<0.01**
Herbivory (%) Second flush	Season	64.40	2	**<0.01**
	Flavonoids	266.50	1	**<0.01**
	Hydroxycinnamic acids	6.18	1	**<0.01**
	Condensed tannins	4.03	1	**0.045**
	Hydrolysable tannins	8.04	1	**<0.01**
	Season × Flavonoids	486.54	2	**<0.01**
	Season × Hydroxycinnamic acids	14.59	2	**<0.01**
	Season × Condensed tannins	12.39	2	**<0.01**
	Season × Hydrolysable tannins	117.64	2	**<0.01**

Table S5. The effect of season, nitrogen, phosphorus and their interactions on the proportion of leaves with leaf mines separately for the first and the second oak leaf flush (*Quercus robur*). Shown are χ^2 values, degrees of freedom and p-values from linear mixed effect models, with significant p-values ($p < 0.05$) in bold.

**Response variable**	**Predictor**	χ^2	**df**	**p**
Proportion of leaves with leaf mines First flush	Season	283.28	2	**<0.01**
	Nitrogen	14.65	1	**<0.01**
	Phosphorus	8.93	1	**<0.01**
	Season × Nitrogen	2.53	2	0.28
	Season × Phosphorus	6.90	2	**0.03**
Proportion of leaves with leaf mines Second flush	Season	38.10	2	**<0.01**
	Nitrogen	8.62	1	**0.03**
	Phosphorus	1.33	1	0.25
	Season × Nitrogen	2.56	2	0.28
	Season × Phosphorus	2.71	2	0.26

Table S6. The effect of season, flavonoids, hydroxycinnamic acids, condensed tannins, hydrolysable tannins and their interactions on the proportion of leaves separately for the first and the second oak leaf flush (*Quercus robur*). Shown are χ^2 values, degrees of freedom and p-values from linear mixed effect models, with significant p-values ($p < 0.05$) in bold.

**Response variable**	**Predictor**	χ^2	**df**	**p**
Proportion of leaves with leaf mines First flush	Season	223.11	2	**<0.01**
	Flavonoids	0.47	1	0.50
	Hydroxycinnamic acids	0.12	1	0.73
	Condensed tannins	11.19	1	**<0.01**
	Hydrolysable tannins	2.44	1	0.12
	Season × Flavonoids	35.49	2	**<0.01**
	Season × Hydroxycinnamic acids	0.94	2	0.626
	Season × Condensed tannins	10.36	2	**<0.01**
	Season × Hydrolysable tannins	16.10	2	**<0.01**
Proportion of leaves with leaf mines Second flush	Season	28.46	2	**<0.01**
	Flavonoids	2.13	1	0.15
	Hydroxycinnamic acids	6.22	1	**0.01**
	Condensed tannins	22.74	1	**<0.01**
	Hydrolysable tannins	15.31	1	**<0.01**
	Season × Flavonoids	29.86	2	**<0.01**
	Season × Hydroxycinnamic acids	2.51	2	0.29
	Season × Condensed tannins	11.24	2	**<0.01**
	Season × Hydrolysable tannins	17.12	2	**<0.01**

Table S7. The effect of season, nitrogen, phosphorus and their interactions on the proportion of leaves with galls separately for the first and the second oak leaf flush (*Quercus robur*). Shown are χ^2 values, degrees of freedom and p-values from linear mixed effect models, with significant p-values ($p < 0.05$) in bold.

**Response variable**	**Predictor**	χ^2	**df**	**p**
Proportion of leaves with galls First flush	Season	108.34	2	**<0.01**
	Nitrogen	0.03	1	0.87
	Phosphorus	7.91	1	**<0.01**
	Season × Nitrogen	1.10	2	0.58
	Season × Phosphorus	5.06	2	0.08
Proportion of leaves with galls Second flush	Season	25.36	2	**<0.01**
	Nitrogen	0.37	1	0.54
	Phosphorus	0.18	1	0.67
	Season × Nitrogen	1.29	2	0.52
	Season × Phosphorus	1.19	2	0.55

Table S8. The effect of season, flavonoids, hydroxycinnamic acids, condensed tannins, hydrolysable tannins and their interactions on the proportion of leaves separately for the first and the second oak leaf flush (*Quercus robur*). Shown are χ^2 values, degrees of freedom and p-values from linear mixed effect models, with significant p-values ($p < 0.05$) in bold.

Response variable	Predictor	χ^2	df	p
Proportion of leaves with galls First flush	Season	104.67	2	**<0.01**
	Flavonoids	0.15	1	0.70
	Hydroxycinnamic acids	0.18	1	0.67
	Condensed tannins	1.30	1	0.26
	Hydrolysable tannins	3.45	1	0.06
	Season × Flavonoids	11.42	2	**<0.01**
	Season × Hydroxycinnamic acids	1.40	2	0.50
	Season × Condensed tannins	27.76	2	**<0.01**
	Season × Hydrolysable tannins	1.27	2	0.53
Proportion of leaves with galls Second flush	Season	16.38	2	**<0.01**
	Flavonoids	3.46	1	0.06
	Hydroxycinnamic acids	4.96	1	**0.03**
	Condensed tannins	0.04	1	0.84
	Hydrolysable tannins	0.38	1	0.54
	Season × Flavonoids	8.32	2	**0.02**
	Season × Hydroxycinnamic acids	2.69	2	0.26
	Season × Condensed tannins	22.31	2	**<0.01**
	Season × Hydrolysable tannins	0.02	2	0.99

Table S9. The effect of seasons, nitrogen, phosphorus and their interactions on the percentage of infection by oak powdery mildew on upper leaf side separately for the first and the second oak leaf flush (*Quercus robur*). Shown are χ^2 values, degrees of freedom and p-values from linear mixed effect models, with significant p-values ($p < 0.05$) in bold.

**Response variable**	**Predictor**	χ^2	**df**	**p**
Infection by oak powdery mildew on upper leaf side (%) First flush	Season	735.64	2	**<0.01**
	Nitrogen	42.59	1	**<0.01**
	Phosphorus	31.97	1	**<0.01**
	Season × Nitrogen	97.43	2	**<0.01**
	Season × Phosphorus	130.29	2	**<0.01**
Infection by oak powdery mildew on upper leaf side (%) Second flush	Season	198.23	2	**<0.01**
	Nitrogen	33.07	1	**<0.01**
	Phosphorus	23.55	1	**<0.01**
	Season × Nitrogen	213.38	2	**<0.01**
	Season × Phosphorus	227.04	2	**<0.01**

Table S10. The effect of season, flavonoids, hydroxycinnamic acids, condensed tannins, hydrolysable tannins and their interactions on the percentage of infection by oak powdery mildew on upper leaf side separately for the first and the second oak leaf flush (*Quercus robur*). Shown are χ^2 values, degrees of freedom and p-values from linear mixed effect models, with significant p-values ($p < 0.05$) in bold.

**Response variable**	**Predictor**	χ^2	**df**	**p**
Infection by oak powdery mildew on upper leaf side (%) First flush	Season	604.03	2	**<0.01**
	Flavonoids	8.80	1	**<0.01**
	Hydroxycinnamic acids	0.18	1	0.67
	Condensed tannins	0.18	1	0.67
	Hydrolysable tannins	0.54	1	0.46
	Season × Flavonoids	139.33	2	**<0.01**
	Season × Hydroxycinnamic acids	112.60	2	**<0.01**
	Season × Condensed tannins	31.98	2	**<0.01**
	Season × Hydrolysable tannins	36.04	2	**<0.01**
Infection by oak powdery mildew on upper leaf side (%) Second flush	Season	370.02	2	**<0.01**
	Flavonoids	2.72	1	0.10
	Hydroxycinnamic acids	79.31	1	**<0.01**
	Condensed tannins	104.84	1	**<0.01**
	Hydrolysable tannins	100.32	1	**<0.01**
	Season × Flavonoids	115.18	2	**<0.01**
	Season × Hydroxycinnamic acids	28.62	2	**<0.01**
	Season × Condensed tannins	23.14	2	**<0.01**
	Season × Hydrolysable tannins	148.40	2	**<0.01**

Table S11. The effect of seasons, nitrogen, phosphorus and their interactions on the percentage of infection by oak powdery mildew on lower leaf side separately for the first and the second oak leaf flush (*Quercus robur*). Shown are χ^2 values, degrees of freedom and p-values from linear mixed effect models, with significant p-values ($p < 0.05$) in bold.

**Response variable**	**Predictor**	χ^2	**df**	**p**
Infection by oak powdery mildew on lower leaf side (%) First flush	Season	519.27	2	**<0.01**
	Nitrogen	17.46	1	**<0.01**
	Phosphorus	8.48	1	**<0.01**
	Season × Nitrogen	53.56	2	**<0.01**
	Season × Phosphorus	15.39	2	**<0.01**
Infection by oak powdery mildew on lower leaf side (%) Second flush	Season	656.92	2	**<0.01**
	Nitrogen	28.71	1	**<0.01**
	Phosphorus	97.40	1	**<0.01**
	Season × Nitrogen	190.97	2	**<0.01**
	Season × Phosphorus	545.62	2	**<0.01**

Table S12. The effect of seasons, flavonoids, hydroxycinnamic acids, condensed tannins, hydrolysable tannins and their interactions on the percentage of infection by oak powdery mildew on lower leaf side separately for the first and the second oak leaf flush (*Quercus robur*). Shown are χ^2 values, degrees of freedom and p-values from linear mixed effect models, with significant p-values ($p < 0.05$) in bold.

**Response variable**	**Predictor**	χ^2	**df**	**p**
Infection by oak powdery mildew on lower leaf side (%) First flush	Season	535.08	2	**<0.01**
	Flavonoids	1.75	1	0.19
	Hydroxycinnamic acids	1.79	1	0.18
	Condensed tannins	11.71	1	**<0.01**
	Hydrolysable tannins	0.96	1	0.33
	Season × Flavonoids	13.10	2	**<0.01**
	Season × Hydroxycinnamic acids	6.34	2	**0.042**
	Season × Condensed tannins	33.00	2	**<0.01**
	Season × Hydrolysable tannins	20.22	2	**<0.01**
Infection by oak powdery mildew on lower leaf side (%) Second flush	Season	148.70	2	**<0.01**
	Flavonoids	180.64	1	**<0.01**
	Hydroxycinnamic acids	2.15	1	0.14
	Condensed tannins	12.34	1	**<0.01**
	Hydrolysable tannins	80.43	1	**<0.01**
	Season × Flavonoids	476.92	2	**<0.01**
	Season × Hydroxycinnamic acids	36.34	2	**<0.01**
	Season × Condensed tannins	22.93	2	**<0.01**
	Season × Hydrolysable tannins	36.86	2	**<0.01**

Table S13. The effect of nitrogen, phosphorus and their interactions on the percentage of herbivory separately for the early, mid and late season in oak leaves (*Quercus robur*). Shown are χ^2 values, degrees of freedom and p-values from linear mixed effect models, with significant p-values ($p < 0.05$) in bold.

**Response variable**	**Predictor**	χ^2	**df**	**p**
Herbivory (%) Early season	Leaf flush	27.92	1	**<0.01**
	Nitrogen	4.04	1	**0.045**
	Phosphorus	<.01	1	0.99
	Leaf flush × Nitrogen	<.01	1	0.92
	Leaf flush × Phosphorus	1.19	1	0.28
Herbivory (%) Mid season	Leaf flush	60.86	2	**<0.01**
	Nitrogen	25.80	1	**<0.01**
	Phosphorus	14.93	1	**<0.01**
	Leaf flush × Nitrogen	26.00	2	**<0.01**
	Leaf flush × Phosphorus	168.89	2	**<0.01**
Herbivory (%) Late season	Leaf flush	72.18	2	**<0.01**
	Nitrogen	2.38	1	0.12
	Phosphorus	47.58	1	**<0.01**
	Leaf flush × Nitrogen	33.23	2	**<0.01**
	Leaf flush × Phosphorus	15.77	2	**<0.01**

Table S14. The effect of oak leaf flush (*Quercus robur*), flavonoids, hydroxycinnamic acids, condensed tannins, hydrolysable tannins and their interactions on the percentage of herbivory separately for the early, mid and late season. Shown are χ^2 values, degrees of freedom and p-values from linear mixed effect models, with significant p-values ($p < 0.05$) in bold.

**Response variable**	**Predictor**	**χ^2**	**df**	**p**
Herbivory (%) Early season	Leaf flush	1.11	1	0.29
	Flavonoids	9.82	1	**<0.01**
	Hydroxycinnamic acids	0.11	1	0.74
	Condensed tannins	9.76	1	**<0.01**
	Hydrolysable tannins	3.38	1	0.07
	Leaf flush × Flavonoids	8.20	1	**<0.01**
	Leaf flush × Hydroxycinnamic acids	1.11	1	0.29
	Leaf flush × Condensed tannins	2.29	1	0.13
	Leaf flush × Hydrolysable tannins	4.19	1	**0.04**
Herbivory (%) Mid season	Leaf flush	33.03	2	**<0.01**
	Flavonoids	0.13	1	0.71
	Hydroxycinnamic acids	<.01	1	0.98
	Condensed tannins	3.83	1	**0.05**
	Hydrolysable tannins	0.84	1	0.36
	Leaf flush × Flavonoids	14.95	2	**<0.01**
	Leaf flush × Hydroxycinnamic acids	10.90	2	**<0.01**
	Leaf flush × Condensed tannins	0.21	2	0.65
	Leaf flush × Hydrolysable tannins	15.72	2	**<0.01**
Herbivory (%) Late season	Leaf flush	145.06	2	**<0.01**
	Flavonoids	<.01	1	0.94
	Hydroxycinnamic acids	2.06	1	0.15
	Condensed tannins	1.33	1	0.25
	Hydrolysable tannins	0.16	1	0.69
	Leaf flush × Flavonoids	17.87	2	**<0.01**
	Leaf flush × Hydroxycinnamic acids	14.70	2	**<0.01**
	Leaf flush × Condensed tannins	47.59	2	**<0.01**
	Leaf flush × Hydrolysable tannins	16.50	2	**<0.01**

Table S15. The effect of nitrogen, phosphorus and their interactions on the proportion of leaves with leaf mines separately for the early, mid and late season in oak leaves (*Quercus robur*). Shown are χ^2 values, degrees of freedom and p-values from linear mixed effect models, with significant p-values ($p < 0.05$) in bold.

Response variable	Predictor	χ^2	df	p
Proportion of leaves with leaf mines Early season	Leaf flush	0.79	1	0.38
	Nitrogen	0.50	1	0.48
	Phosphorus	0.76	1	0.38
	Leaf flush × Nitrogen	0.04	1	0.84
	Leaf flush × Phosphorus	0.34	1	0.56
Proportion of leaves with leaf mines Mid season	Leaf flush	245.30	2	**<0.01**
	Nitrogen	32.91	1	**<0.01**
	Phosphorus	2.55	1	0.11
	Leaf flush × Nitrogen	3.39	2	0.18
	Leaf flush × Phosphorus	13.76	2	**<0.01**
Proportion of leaves with leaf mines Late season	Leaf flush	119.48	2	**<0.01**
	Nitrogen	0.86	1	0.35
	Phosphorus	0.87	1	0.35
	Leaf flush × Nitrogen	1.95	2	0.38
	Leaf flush × Phosphorus	9.60	2	**<0.01**

Table S16. The effect of oak leaf flush (*Quercus robur*), flavonoids, hydroxycinnamic acids, condensed tannins, hydrolysable tannins and their interactions on the proportion of leaves with leaf mines separately for the early, mid and late season. Shown are χ^2 values, degrees of freedom and p-values from linear mixed effect models, with significant p-values ($p < 0.05$) in bold.

**Response variable**	**Predictor**	χ^2	**df**	**p**
Proportion of leaves with leaf mines Early season	Leaf flush	0.50	1	0.48
	Flavonoids	<.01	1	0.99
	Hydroxycinnamic acids	<.01	1	0.96
	Condensed tannins	0.27	1	0.61
	Hydrolysable tannins	0.12	1	0.73
	Leaf flush × Flavonoids	2.66	1	0.10
	Leaf flush × Hydroxycinnamic acids	0.59	1	0.44
	Leaf flush × Condensed tannins	0.47	1	0.50
	Leaf flush × Hydrolysable tannins	<.01	1	0.96
Proportion of leaves with leaf mines Mid season	Leaf flush	269.19	2	**<0.01**
	Flavonoids	0.940	1	0.33
	Hydroxycinnamic acids	0.162	1	0.69
	Condensed tannins	8.623	1	**<0.01**
	Hydrolysable tannins	0.537	1	0.46
	Leaf flush × Flavonoids	37.58	2	**<0.01**
	Leaf flush × Hydroxycinnamic acids	2.55	2	0.28
	Leaf flush × Condensed tannins	3.17	2	0.08
	Leaf flush × Hydrolysable tannins	16.04	2	**<0.01**
Proportion of leaves with leaf mines Late season	Leaf flush	173.07	2	**<0.01**
	Flavonoids	4.30	1	**0.04**
	Hydroxycinnamic acids	2.38	1	0.12
	Condensed tannins	3.18	1	0.08
	Hydrolysable tannins	<.01	1	0.94
	Leaf flush × Flavonoids	12.20	2	**<0.01**
	Leaf flush × Hydroxycinnamic acids	4.37	2	0.11
	Leaf flush × Condensed tannins	5.32	2	0.07
	Leaf flush × Hydrolysable tannins	4.11	2	0.13

Table S17. The effect of nitrogen, phosphorus and their interactions on the proportion of leaves with galls separately for the early, mid and late season in oak leaves (*Quercus robur*). Shown are χ^2 values, degrees of freedom and p-values from linear mixed effect models, with significant p-values ($p < 0.05$) in bold.

Response variable	Predictor	χ^2	df	p
Proportion of leaves with galls Early season	Leaf flush	1.14	1	0.29
	Nitrogen	4.34	1	**0.04**
	Phosphorus	8.53	1	**<0.01**
	Leaf flush × Nitrogen	0.89	1	0.35
	Leaf flush × Phosphorus	7.63	1	**<0.01**
Proportion of leaves with galls Mid season	Leaf flush	123.66	2	**<0.01**
	Nitrogen	6.17	1	**0.01**
	Phosphorus	7.42	1	**<0.01**
	Leaf flush × Nitrogen	5.53	2	0.06
	Leaf flush × Phosphorus	0.29	2	0.86
Proportion of leaves with leaf galls Late season	Leaf flush	16.69	2	**<0.01**
	Nitrogen	3.94	1	**0.047**
	Phosphorus	0.01	1	0.92
	Leaf flush × Nitrogen	1.82	2	0.40
	Leaf flush × Phosphorus	1.66	2	0.44

Table S18. The effect of oak leaf flush (*Quercus robur*), flavonoids, hydroxycinnamic acids, condensed tannins, hydrolysable tannins and their interactions on the proportion of leaves with galls separately for the early, mid and late season. Shown are χ^2 values, degrees of freedom and p-values from linear mixed effect models, with significant p-values ($p < 0.05$) in bold.

**Response variable**	**Predictor**	χ^2	**df**	**p**
Proportion of leaves with galls Early season	Leaf flush	0.75	1	0.39
	Flavonoids	1.57	1	0.21
	Hydroxycinnamic acids	1.54	1	0.22
	Condensed tannins	1.40	1	0.24
	Hydrolysable tannins	0.43	1	0.51
	Leaf flush × Flavonoids	7.52	1	**<0.01**
	Leaf flush × Hydroxycinnamic acids	1.68	1	0.20
	Leaf flush × Condensed tannins	0.27	1	0.61
	Leaf flush × Hydrolysable tannins	8.36	1	**<0.01**
Proportion of leaves with galls Mid season	Leaf flush	21.98	2	**<0.01**
	Flavonoids	19.81	1	**<0.01**
	Hydroxycinnamic acids	0.01	1	0.91
	Condensed tannins	37.11	1	**<0.01**
	Hydrolysable tannins	0.27	1	0.61
	Leaf flush × Flavonoids	46.48	2	**<0.01**
	Leaf flush × Hydroxycinnamic acids	16.12	2	**<0.01**
	Leaf flush × Condensed tannins	0.90	2	0.34
	Leaf flush × Hydrolysable tannins	9.38	2	**<0.01**
Proportion of leaves with galls Late season	Leaf flush	53.82	2	**<0.01**
	Flavonoids	0.32	1	0.57
	Hydroxycinnamic acids	0.06	1	0.81
	Condensed tannins	3.67	1	0.06
	Hydrolysable tannins	0.28	1	0.60
	Leaf flush × Flavonoids	2.94	2	0.23
	Leaf flush × Hydroxycinnamic acids	5.16	2	0.08
	Leaf flush × Condensed tannins	1.08	2	0.58
	Leaf flush × Hydrolysable tannins	3.58	2	0.17

Table S19. The effect of nitrogen, phosphorus and their interactions on the percentage of infection by oak powdery mildew on upper leaf separately for the early, mid and late season in oak leaves (*Quercus robur*). Shown are χ^2 values, degrees of freedom and p-values from linear mixed effect models, with significant p-values ($p < 0.05$) in bold.

**Response variable**	**Predictor**	χ^2	**df**	**p**
Infection by oak powdery mildew on upper leaf side (%) Early season	Leaf flush	1778.97	1	**<0.01**
	Nitrogen	23.71	1	**<0.01**
	Phosphorus	23.81	1	**<0.01**
	Leaf flush × Nitrogen	42.78	1	**<0.01**
	Leaf flush × Phosphorus	30.45	1	**<0.01**
Infection by oak powdery mildew on upper leaf side (%) Mid season	Leaf flush	450.72	2	**<0.01**
	Nitrogen	88.03	1	**<0.01**
	Phosphorus	14.96	1	**<0.01**
	Leaf flush × Nitrogen	61.87	2	**<0.01**
	Leaf flush × Phosphorus	78.82	2	**<0.01**
Infection by oak powdery mildew on upper leaf side (%) Late season	Leaf flush	118.53	2	**<0.01**
	Nitrogen	102.85	1	**<0.01**
	Phosphorus	777.96	1	**<0.01**
	Leaf flush × Nitrogen	1.14	2	0.57
	Leaf flush × Phosphorus	323.63	2	**<0.01**

Table S20. The effect of oak leaf flush (*Quercus robur*), flavonoids, hydroxycinnamic acids, condensed tannins, hydrolysable tannins and their interactions on the percentage of infection by oak powdery mildew on upper leaf side separately for the early, mid and late season. Shown are χ^2 values, degrees of freedom and p-values from linear mixed effect models, with significant p-values ($p < 0.05$) in bold.

**Response variable**	**Predictor**	χ^2	**df**	**p**
Infection by oak powdery mildew on upper leaf side (%) Early season	Leaf flush	138.32	1	**<0.01**
	Flavonoids	41.88	1	**<0.01**
	Hydroxycinnamic acids	79.93	1	**<0.01**
	Condensed tannins	0.26	1	0.61
	Hydrolysable tannins	5.07	1	**0.02**
	Leaf flush × Flavonoids	121.17	1	**<0.01**
	Leaf flush × Hydroxycinnamic acids	2.73	1	0.10
	Leaf flush × Condensed tannins	7.28	1	**0.01**
	Leaf flush × Hydrolysable tannins	13.80	1	**<0.01**
Infection by oak powdery mildew on upper leaf side (%) Mid season	Leaf flush	859.85	2	**<0.01**
	Flavonoids	0.28	1	0.60
	Hydroxycinnamic acids	23.23	1	**<0.01**
	Condensed tannins	62.24	1	**<0.01**
	Hydrolysable tannins	86.65	1	**<0.01**
	Leaf flush × Flavonoids	44.96	2	**<0.01**
	Leaf flush × Hydroxycinnamic acids	51.54	2	**<0.01**
	Leaf flush × Condensed tannins	<.01	2	0.93
	Leaf flush × Hydrolysable tannins	0.68	2	0.41
Infection by oak powdery mildew on upper leaf side (%) Late season	Leaf flush	1834.36	2	**<0.01**
	Flavonoids	6.05	1	**0.01**
	Hydroxycinnamic acids	60.16	1	**<0.01**
	Condensed tannins	95.37	1	**<0.01**
	Hydrolysable tannins	21.80	1	**<0.01**
	Leaf flush × Flavonoids	95.85	2	**<0.01**
	Leaf flush × Hydroxycinnamic acids	69.41	2	**<0.01**
	Leaf flush × Condensed tannins	25.36	2	**<0.01**
	Leaf flush × Hydrolysable tannins	472.94	2	**<0.01**

Table S21. The effect of nitrogen, phosphorus and their interactions on the percentage of infection by oak powdery mildew on lower leaf side separately for the early, mid and late season in oak leaves (*Quercus robur*). Shown are χ^2 values, degrees of freedom and p-values from linear mixed effect models, with significant p-values ($p < 0.05$) in bold.

**Response variable**	**Predictor**	χ^2	**df**	**p**
Infection by oak powdery mildew on lower leaf side (%) Early season	Leaf flush	2.67	1	0.10
	Nitrogen	6.57	1	**0.01**
	Phosphorus	1.24	1	0.27
	Leaf flush × Nitrogen	61.73	1	**<0.01**
	Leaf flush × Phosphorus	10.15	1	**<0.01**
Infection by oak powdery mildew on lower leaf side (%) Mid season	Leaf flush	264.46	2	**<0.01**
	Nitrogen	23.36	1	**<0.01**
	Phosphorus	31.35	1	**<0.01**
	Leaf flush × Nitrogen	13.65	2	**<0.01**
	Leaf flush × Phosphorus	4.72	2	0.09
Infection by oak powdery mildew on lower leaf side (%) Late season	Leaf flush	36.75	2	**<0.01**
	Nitrogen	7.74	1	**<0.05**
	Phosphorus	935.84	1	**<0.01**
	Leaf flush × Nitrogen	137.92	2	**<0.01**
	Leaf flush × Phosphorus	319.88	2	**<0.01**

Table S22. The effect of oak leaf flush (*Quercus robur*), flavonoids, hydroxycinnamic acids, condensed tannins, hydrolysable tannins and their interactions on the percentage of infection by oak powdery mildew on lower leaf side separately for the early, mid and late season. Shown are χ^2 values, degrees of freedom and p-values from linear mixed effect models, with significant p-values ($p < 0.05$) in bold.

Response variable	Predictor	χ^2	df	p
Infection by oak powdery mildew on lower leaf side (%) Early season	Leaf flush	20.02	1	**<0.01**
	Flavonoids	47.18	1	**<0.01**
	Hydroxycinnamic acids	1.20	1	0.27
	Condensed tannins	9.90	1	**<0.01**
	Hydrolysable tannins	0.08	1	0.78
	Leaf flush × Flavonoids	19.24	1	**<0.01**
	Leaf flush × Hydroxycinnamic acids	1.21	1	0.27
	Leaf flush × Condensed tannins	46.57	1	**<0.01**
	Leaf flush × Hydrolysable tannins	3.51	1	0.06
Infection by oak powdery mildew on lower leaf side (%) Mid season	Leaf flush	581.66	2	**<0.01**
	Flavonoids	56.46	1	**<0.01**
	Hydroxycinnamic acids	5.85	1	**0.02**
	Condensed tannins	4.39	1	**0.04**
	Hydrolysable tannins	3.27	1	0.07
	Leaf flush × Flavonoids	57.12	2	**<0.01**
	Leaf flush × Hydroxycinnamic acids	34.84	2	**<0.01**
	Leaf flush × Condensed tannins	1.27	2	0.26
	Leaf flush × Hydrolysable tannins	2.06	2	0.15
Infection by oak powdery mildew on lower leaf side (%) Late season	Leaf flush	275.42	2	**<0.01**
	Flavonoids	2.44	1	0.12
	Hydroxycinnamic acids	2.42	1	0.12
	Condensed tannins	109.18	1	**<0.01**
	Hydrolysable tannins	16.13	1	**<0.01**
	Leaf flush × Flavonoids	582.01	2	**<0.01**
	Leaf flush × Hydroxycinnamic acids	13.79	2	**<0.01**
	Leaf flush × Condensed tannins	93.92	2	**<0.01**
	Leaf flush × Hydrolysable tannins	221.60	2	**<0.01**

Table S23. The impact of latitude on the relative abundance of the first, second and third oak leaf flush (*Quercus robur*). To account for the hierarchical and repeated-measures design, we included country, population and tree identity as random intercepts. Shown are χ^2 values, degrees of freedom and p-values from a linear mixed effect model, with significant p-values ($p < 0.05$) in bold.

**Response variable**	**Predictor**	**χ^2**	**df**	**p**
Leaf flush	Latitude	16.52	1	**<0.01**
	Season	15.70	2	**<0.01**

Table S24. Results of paired tests comparing the concentration of primary (nitrogen, phosphorus), secondary compounds (flavonoids, hydroxycinnamic acids, condensed tannins and hydrolysable tannins), insect attack (percentage of free-feeding herbivory (%), proportion of leaves with leaf mines, proportion of leaves with galls) and pathogen infection (percentage of infection by powdery mildew on upper and lower leaf side) among seasons in oak leaves (*Quercus robur*). Shown are the groups compared, estimates, standard errors (SE), t-ratios and p-values. Significant p-values ($p < 0.05$) shown in bold.

**Response variable**	**Flush**	**Contrast**	**Estimate**	**SE**	**t-ratio**	**p**
Nitrogen	First	Early – Mid	1.61	0.21	7.78	**<0.001**
Nitrogen	First	Early – Late	6.57	0.21	31.83	**<0.001**
Nitrogen	First	Mid – Late	4.97	0.21	24.05	**<0.001**
Nitrogen	Second	Early – Mid	2.57	0.22	11.78	**<0.001**
Nitrogen	Second	Early – Late	2.32	0.22	10.52	**<0.001**
Nitrogen	Second	Mid – Late	-0.25	0.17	-1.45	0.314
Nitrogen	Third	Mid – Late	4.62	0.58	7.99	**<0.001**
Phosphorus	First	Early – Mid	0.53	0.01	38.56	**<0.001**
Phosphorus	First	Early – Late	0.21	0.01	15.03	**<0.001**
Phosphorus	First	Mid – Late	-0.32	0.01	-23.52	**<0.001**
Phosphorus	Second	Early – Mid	0.65	0.01	50.43	**<0.001**
Phosphorus	Second	Early – Late	0.47	0.01	36.33	**<0.001**
Phosphorus	Second	Mid – Late	-0.18	0.01	-17.64	**<0.001**
Phosphorus	Third	Mid – Late	0.71	0.03	21.62	**<0.001**
Flavonoids	First	Early – Mid	-0.49	0.23	-2.20	0.072
Flavonoids	First	Early – Late	4.97	0.23	22.12	**<0.001**
Flavonoids	First	Mid – Late	5.47	0.23	24.32	**<0.001**
Flavonoids	Second	Early – Mid	6.92	0.60	11.62	**<0.001**
Flavonoids	Second	Early – Late	21.77	0.60	36.07	**<0.001**
Flavonoids	Second	Mid – Late	14.85	0.46	32.25	**<0.001**
Flavonoids	Third	Mid – Late	24.4	0.86	28.56	**<0.001**
Hydroxycinnamic acids	First	Early – Mid	-0.18	0.08	-2.30	**<0.001**
Hydroxycinnamic acids	First	Early – Late	2.20	0.08	28.44	**<0.001**
Hydroxycinnamic acids	First	Mid – Late	2.38	0.08	30.74	**<0.001**
Hydroxycinnamic acids	Second	Early – Mid	1.21	0.14	8.69	**<0.001**
Hydroxycinnamic acids	Second	Early – Late	-0.12	0.14	-0.88	0.656
Hydroxycinnamic acids	Second	Mid – Late	1.33	0.11	12.52	**<0.001**
Hydroxycinnamic acids	Third	Mid – Late	4.96	0.28	17.69	**<0.001**
Condensed tannins	First	Early – Mid	-0.70	0.10	-7.15	**<0.001**
Condensed tannins	First	Early – Late	-2.24	0.10	-22.89	**<0.001**
Condensed tannins	First	Mid – Late	-1.54	0.10	-15.73	**<0.001**
Condensed tannins	Second	Early – Mid	-0.04	0.28	-0.16	0.987
Condensed tannins	Second	Early – Late	3.69	0.28	13.12	**<0.001**
Condensed tannins	Second	Mid – Late	3.74	0.22	17.40	**<0.001**
Condensed tannins	Third	Mid – Late	-0.48	0.27	-1.73	0.085
Hydrolysable tannins	First	Early – Mid	-0.39	0.03	-11.78	**<0.001**
Hydrolysable tannins	First	Early – Late	-0.02	0.03	-0.48	0.880
Hydrolysable tannins	First	Mid – Late	0.37	0.03	1.30	0.841
Hydrolysable tannins	Second	Early – Mid	1.05	0.10	10.06	**<0.001**
Hydrolysable tannins	Second	Early – Late	3.59	0.11	34.10	**<0.001**
Hydrolysable tannins	Second	Mid – Late	2.55	0.08	31.69	**<0.001**
Hydrolysable tannins	Third	Mid – Late	3.41	0.07	51.42	**<0.001**
Herbivory (%)	First	Early – Mid	-1.73	0.31	-5.51	**<0.001**
Herbivory (%)	First	Early – Late	-3.28	0.31	-10.45	**<0.001**
Herbivory (%)	First	Mid – Late	-1.55	0.32	-4.93	**<0.001**
Herbivory (%)	Second	Early – Mid	-4.58	0.48	-9.53	**<0.001**
Herbivory (%)	Second	Early – Late	-5.29	0.49	-10.86	**<0.001**
Herbivory (%)	Second	Mid – Late	-0.71	0.37	-1.91	0.135
Herbivory (%)	Third	Mid – Late	-0.02	0.16	-0.14	0.888

Proportion of leaves with leaf mines	First	Early – Mid	-0.13	0.01	-11.83	**<0.001**
Proportion of leaves with leaf mines	First	Early – Late	-0.20	0.01	-16.99	**<0.001**
Proportion of leaves with leaf mines	First	Mid – Late	-0.06	0.01	-5.15	**<0.001**
Proportion of leaves with leaf mines	Second	Early – Mid	-0.07	0.01	-5.74	**<0.001**
Proportion of leaves with leaf mines	Second	Early – Late	-0.09	0.01	-7.53	**<0.001**
Proportion of leaves with leaf mines	Second	Mid – Late	-0.02	0.01	-2.47	**0.036**
Proportion of leaves with leaf mines	Third	Mid – Late	0.02	0.01	1.27	0.218
Proportion of leaves with galls	First	Early – Mid	-0.05	0.01	-4.85	**<0.001**
Proportion of leaves with galls	First	Early – Late	-0.11	0.01	-11.94	**<0.001**
Proportion of leaves with galls	First	Mid – Late	-0.07	0.01	-7.07	**<0.001**
Proportion of leaves with galls	Second	Early – Mid	-0.01	0.01	-2.28	0.060
Proportion of leaves with galls	Second	Early – Late	-0.03	0.01	-4.91	**<0.001**
Proportion of leaves with galls	Second	Mid – Late	-0.02	<0.01	-3.55	**0.001**
Proportion of leaves with galls	Third	Mid – Late	<0.01	0.01	-0.03	0.976
Infection by powdery mildew on upper leaf side (%)	First	Early – Mid	-19.24	0.84	-22.80	**<0.001**
Infection by powdery mildew on upper leaf side (%)	First	Early – Late	-13.37	0.84	-15.84	**<0.001**
Infection by powdery mildew on upper leaf side (%)	First	Mid – Late	5.87	0.84	6.96	**<0.001**
Infection by powdery mildew on upper leaf side (%)	Second	Early – Mid	-15.26	1.14	-13.39	**<0.001**
Infection by powdery mildew on upper leaf side (%)	Second	Early – Late	-8.55	1.16	-7.61	**<0.001**
Infection by powdery mildew on upper leaf side (%)	Second	Mid – Late	6.70	0.88	7.61	**<0.001**
Infection by powdery mildew on upper leaf side (%)	Third	Mid – Late	-15.90	4.68	-3.40	**<0.001**
Infection by powdery mildew on lower leaf side (%)	First	Early – Mid	-3.68	0.41	-8.90	**<0.001**
Infection by powdery mildew on lower leaf side (%)	First	Early – Late	-10.58	0.41	-25.57	**<0.001**
Infection by powdery mildew on lower leaf side (%)	First	Mid – Late	-6.90	0.41	-16.66	**<0.001**
Infection by powdery mildew on lower leaf side (%)	Second	Early – Mid	-11.30	1.57	-7.19	**<0.001**
Infection by powdery mildew on lower leaf side (%)	Second	Early – Late	-28.40	1.59	-17.83	**<0.001**
Infection by powdery mildew on lower leaf side (%)	Second	Mid – Late	-17.10	1.21	-14.09	**<0.001**
Infection by powdery mildew on lower leaf side (%)	Third	Mid – Late	-28.8	2.65	-10.88	**<0.001**

Table S25. Results of paired tests comparing the concentration of primary (nitrogen, phosphorus), secondary compounds (flavonoids, hydroxycinnamic acids, condensed tannins and hydrolysable tannins), insect attack (percentage of free-feeding herbivory (%), proportion of leaves with leaf mines, proportion of leaves with galls) and pathogen infection (percentage of infection by powdery mildew on upper and lower leaf side) among leaf flushes during the early, mid and late seasons in oak leaves (*Quercus robur*). Shown are the groups compared, estimates, standard errors (SE), t-ratios and p-values. Significant p-values ($p < 0.05$) shown in bold.

**Response variable**	**Season**	**Contrast**	**Estimate**	**SE**	**t-ratio**	**p**
Nitrogen	Early	First – Second	6.97	0.11	61.34	**<0.001**
Nitrogen	Mid	First – Second	10.64	0.19	57.44	**<0.001**
Nitrogen	Mid	First – Third	3.06	0.63	4.82	**<0.001**
Nitrogen	Mid	Second - Third	-7.58	0.63	-11.95	**<0.001**
Nitrogen	Late	First – Second	6.87	0.12	56.88	**<0.001**
Nitrogen	Late	First – Third	-1.07	0.23	-2.31	**0.048**
Nitrogen	Late	Second - Third	-6.94	0.23	-29.80	**<0.001**
Phosphorus	Early	First – Second	-0.28	0.01	-29.98	**<0.001**
Phosphorus	Mid	First – Second	0.21	<0.01	21.03	**<0.001**
Phosphorus	Mid	First – Third	-0.57	0.03	-16.80	**<0.001**
Phosphorus	Mid	Second - Third	-0.78	0.03	-22.93	**<0.001**
Phosphorus	Late	First – Second	0.47	0.01	61.27	**<0.001**
Phosphorus	Late	First – Third	0.16	0.01	10.88	**<0.001**
Phosphorus	Late	Second - Third	-0.31	0.02	-21.22	**<0.001**
Flavonoids	Early	First – Second	-18.90	0.36	-52.83	**<0.001**
Flavonoids	Mid	First – Second	-11.80	0.34	-35.05	**<0.001**
Flavonoids	Mid	First – Third	-23.80	1.16	-20.63	**<0.001**
Flavonoids	Mid	Second - Third	-12.00	1.16	-10.40	**<0.001**
Flavonoids	Late	First – Second	-2.60	0.24	-10.91	**<0.001**
Flavonoids	Late	First – Third	1.24	0.45	2.78	**0.015**
Flavonoids	Late	Second - Third	3.84	0.46	8.35	**<0.001**
Hydroxycinnamic acids	Early	First – Second	0.62	0.07	8.56	**<0.001**
Hydroxycinnamic acids	Mid	First – Second	1.62	0.09	18.71	**<0.001**
Hydroxycinnamic acids	Mid	First – Third	-2.03	0.30	-6.86	**<0.001**
Hydroxycinnamic acids	Mid	Second - Third	-3.65	0.30	-12.32	**<0.001**
Hydroxycinnamic acids	Late	First – Second	0.60	0.05	12.09	**<0.001**
Hydroxycinnamic acids	Late	First – Third	1.10	0.09	12.79	**<0.001**
Hydroxycinnamic acids	Late	Second - Third	0.50	0.09	5.62	**<0.001**
Condensed tannins	Early	First – Second	-5.39	0.14	-38.38	**<0.001**
Condensed tannins	Mid	First – Second	-8.18	0.19	-43.57	**<0.001**
Condensed tannins	Mid	First – Third	-2.52	0.64	-3.91	**<0.001**
Condensed tannins	Mid	Second - Third	5.67	0.64	8.81	**<0.001**
Condensed tannins	Late	First – Second	-2.45	0.13	-18.99	**<0.001**
Condensed tannins	Late	First – Third	-0.28	0.24	-1.16	**<0.001**
Condensed tannins	Late	Second - Third	2.17	0.25	8.72	**<0.001**
Hydrolysable tannins	Early	First – Second	-4.08	0.05	-86.78	**<0.001**
Hydrolysable tannins	Mid	First – Second	-2.61	0.07	-37.87	**<0.001**
Hydrolysable tannins	Mid	First – Third	-3.04	0.24	-12.88	**<0.001**
Hydrolysable tannins	Mid	Second - Third	-0.43	0.24	-1.83	**<0.001**
Hydrolysable tannins	Late	First – Second	-0.40	0.04	-10.17	**<0.001**
Hydrolysable tannins	Late	First – Third	0.21	0.07	2.78	**<0.001**
Hydrolysable tannins	Late	Second - Third	0.61	0.08	7.97	**<0.001**
Herbivory (%)	Early	First – Second	2.48	0.36	6.94	**<0.001**
Herbivory (%)	Mid	First – Second	3.55	0.38	9.10	**<0.001**
Herbivory (%)	Mid	First – Third	9.49	1.34	7.11	**<0.001**
Herbivory (%)	Mid	Second - Third	5.95	1.34	4.45	**<0.001**
Herbivory (%)	Late	First – Second	5.41	0.53	10.20	**<0.001**
Herbivory (%)	Late	First – Third	9.69	0.99	9.79	**<0.001**
Herbivory (%)	Late	Second - Third	4.27	1.02	4.18	**<0.001**

Proportion of leaves with leaf mines	Early	First – Second	0.33	0.01	11.96	**<0.001**
Proportion of leaves with leaf mines	Mid	First – Second	0.22	0.01	16.61	**<0.001**
Proportion of leaves with leaf mines	Mid	First – Third	0.21	0.04	4.82	**<0.001**
Proportion of leaves with leaf mines	Mid	Second - Third	<0.01	0.04	-0.04	0.999
Proportion of leaves with leaf mines	Late	First – Second	0.19	0.02	11.49	**<0.001**
Proportion of leaves with leaf mines	Late	First – Third	0.24	0.03	8.01	**<0.001**
Proportion of leaves with leaf mines	Late	Second - Third	0.16	0.03	7.75	**0.008**
Proportion of leaves with galls	Early	First – Second	0.03	0.01	2.25	**0.025**
Proportion of leaves with galls	Mid	First – Second	0.10	0.01	10.97	**<0.001**
Proportion of leaves with galls	Mid	First – Third	0.06	0.03	1.61	**0.040**
Proportion of leaves with galls	Mid	Second - Third	-0.05	0.03	-1.59	**0.049**
Proportion of leaves with galls	Late	First – Second	0.09	0.01	7.07	**<0.001**
Proportion of leaves with galls	Late	First – Third	0.08	0.02	3.29	**0.003**
Proportion of leaves with galls	Late	Second - Third	-0.01	0.03	-0.49	0.874
Infection by powdery mildew on upper leaf side (%)	Early	First – Second	-28.60	1.30	-22.07	**<0.001**
Infection by powdery mildew on upper leaf side (%)	Mid	First – Second	-44.70	1.07	-41.73	**<0.001**
Infection by powdery mildew on upper leaf side (%)	Mid	First – Third	-25.10	3.67	-6.83	**<0.001**
Infection by powdery mildew on upper leaf side (%)	Mid	Second - Third	19.60	3.67	5.35	**<0.001**
Infection by powdery mildew on upper leaf side (%)	Late	First – Second	-53.40	1.28	-41.73	**<0.001**
Infection by powdery mildew on upper leaf side (%)	Late	First – Third	-32.10	2.39	-13.44	**<0.001**
Infection by powdery mildew on upper leaf side (%)	Late	Second - Third	21.20	2.47	8.61	**<0.001**
Infection by powdery mildew on lower leaf side (%)	Early	First – Second	-1.50	0.19	-7.92	**<0.001**
Infection by powdery mildew on lower leaf side (%)	Mid	First – Second	-11.50	0.57	-20.09	**<0.001**
Infection by powdery mildew on lower leaf side (%)	Mid	First – Third	12.80	1.96	6.54	**<0.001**
Infection by powdery mildew on lower leaf side (%)	Mid	Second - Third	24.30	1.96	12.41	**<0.001**
Infection by powdery mildew on lower leaf side (%)	Late	First – Second	-23.13	1.13	-20.48	**<0.001**
Infection by powdery mildew on lower leaf side (%)	Late	First – Third	-14.90	2.11	-7.07	**<0.001**
Infection by powdery mildew on lower leaf side (%)	Late	Second - Third	8.24	2.18	3.79	**<0.001**

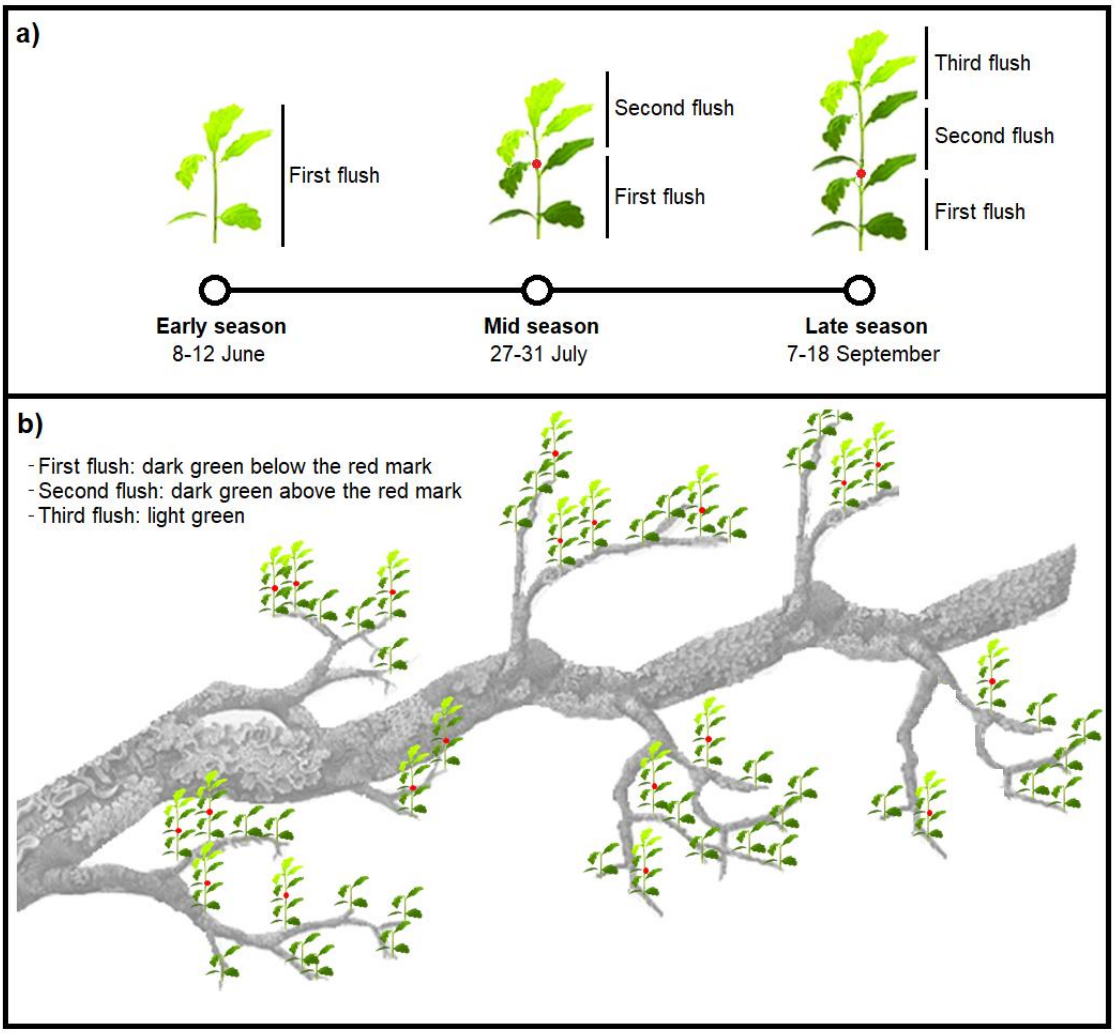

Figure S1. An example to illustrate how we distinguished leaves from different oak leaf flushes (*Quercus robur*), and to clarify our sampling strategy where we randomly sampled leaves within the subset of leaves belonging to a given flush. In the scenario depicted, the tree has one leaf flush during the early season, two leaf flushes during the mid season and three leaf flushes during the late season. Panel **a** illustrates the approach to distinguish between leaf flushes in each season. During the early season, only a single leaf flush was present. During the mid season, we could easily recognize leaves from the first and second leaf flush, as they differ in their shade of green. At this time point, we marked the places where the first leaf flush ends (in red) and the second leaf flush begins with coloured cable ties. During the late season, we could distinguish leaves belonging to the first and second flush with the help of the coloured cable ties, and we could recognize leaves from the third leaf flush by the lighter shade of green. In some locations, where the second leaf flush is only produced during the late part of the season, it was unnecessary to use cable ties. We do note that the cable ties were used to facilitate and speed up the process of distinguishing between the leaf flushes, as the leaf

flushes can also be easily (though not as quickly) distinguished anatomically by the nodes. Panel **b** illustrates an oak branch matching the late-season scenario depicted in panel a, where we randomly sampled: i) 15 leaves from the subset of leaves belonging to the first flush, ii) 15 leaves from the subset of leaves belonging to the second flush and iii) 15 leaves from the subset of leaves belonging to the third flush. Note that for each tree, we repeated this sampling for two branches, resulting in a total of 30 leaves from each leaf flush.

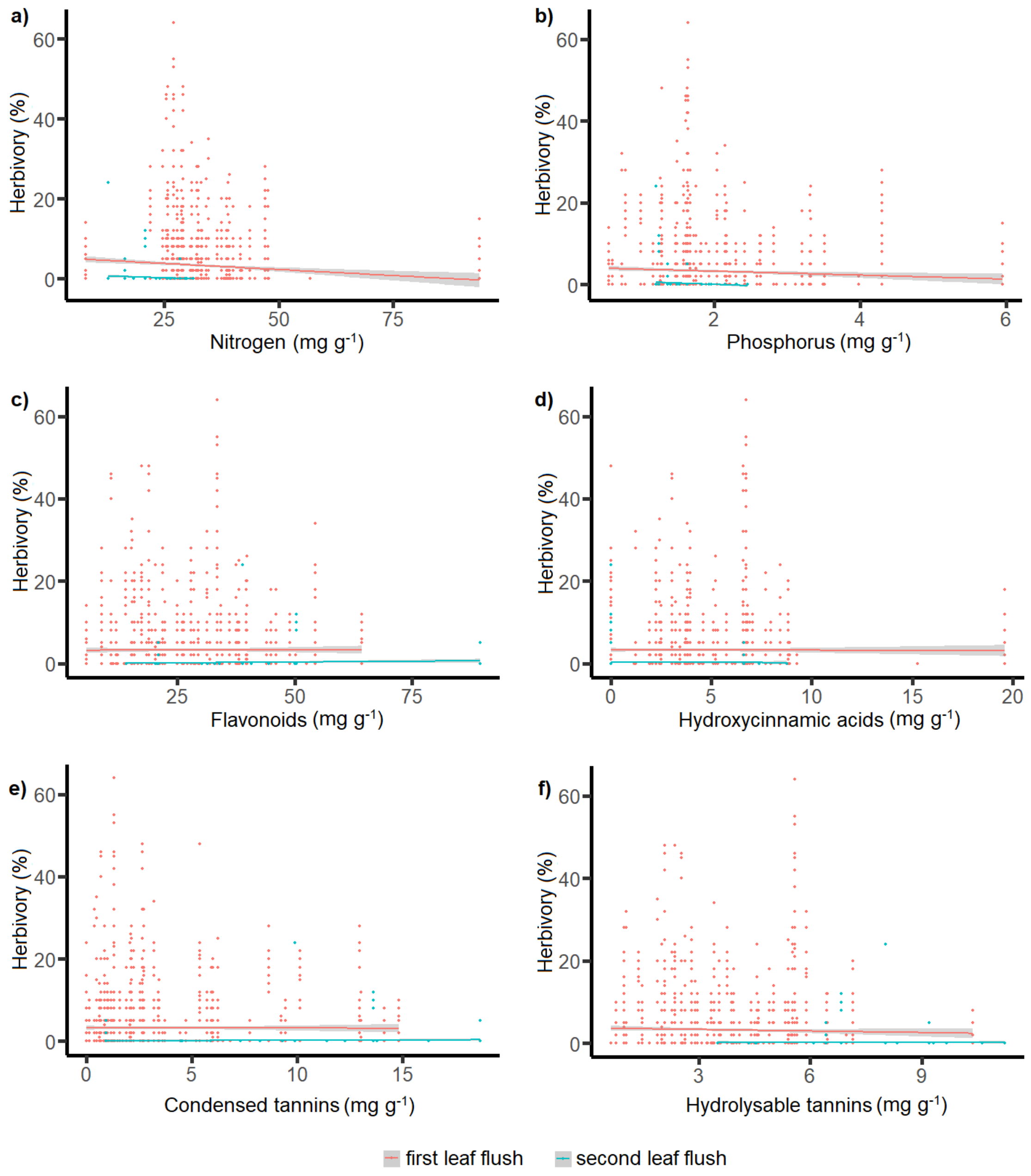

Figure S2. The effect of concentrations of **a)** nitrogen, **b)** phosphorus, **c)** flavonoids, **d)** hydroxycinnamic acids, **e)** condensed tannins and **f)** hydrolysable tannins on the percentage of free-feeding herbivory during the early season in oak leaves (*Quercus robur*). Shown are regression lines with their associated standard error (shaded area), separately for each leaf flush. Concentrations are expressed in mg g^{-1} of dry tissue.

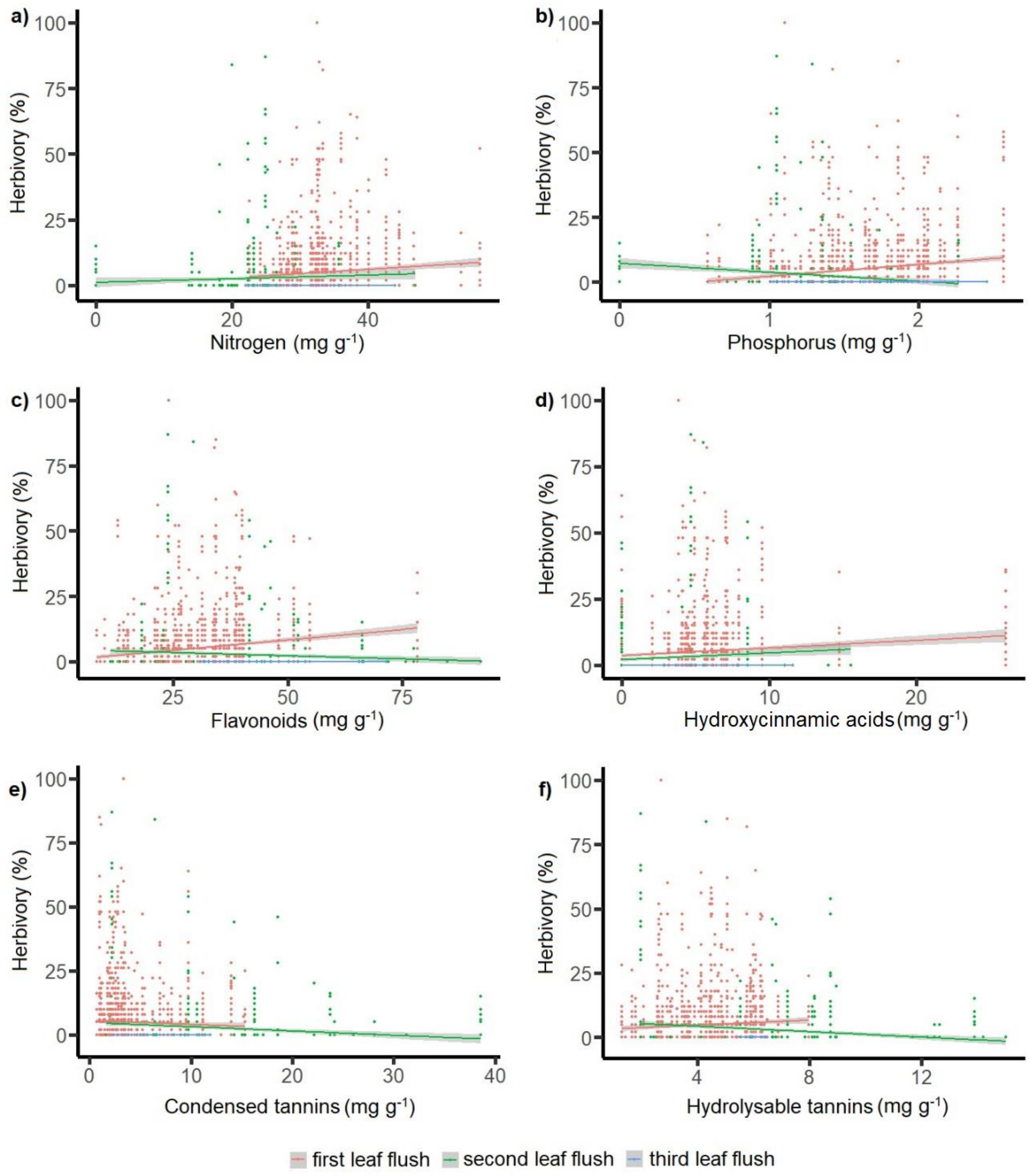

Figure S3. The effect of concentrations of **a)** nitrogen, **b)** phosphorus, **c)** flavonoids, **d)** hydroxycinnamic acids, **e)** condensed tannins and **f)** hydrolysable tannins on the percentage of free-feeding herbivory during the mid season in oak leaves (*Quercus robur*). Shown are regression lines with their associated standard error (shaded area), separately for each leaf flush. Concentrations are expressed in mg g−1 of dry tissue.

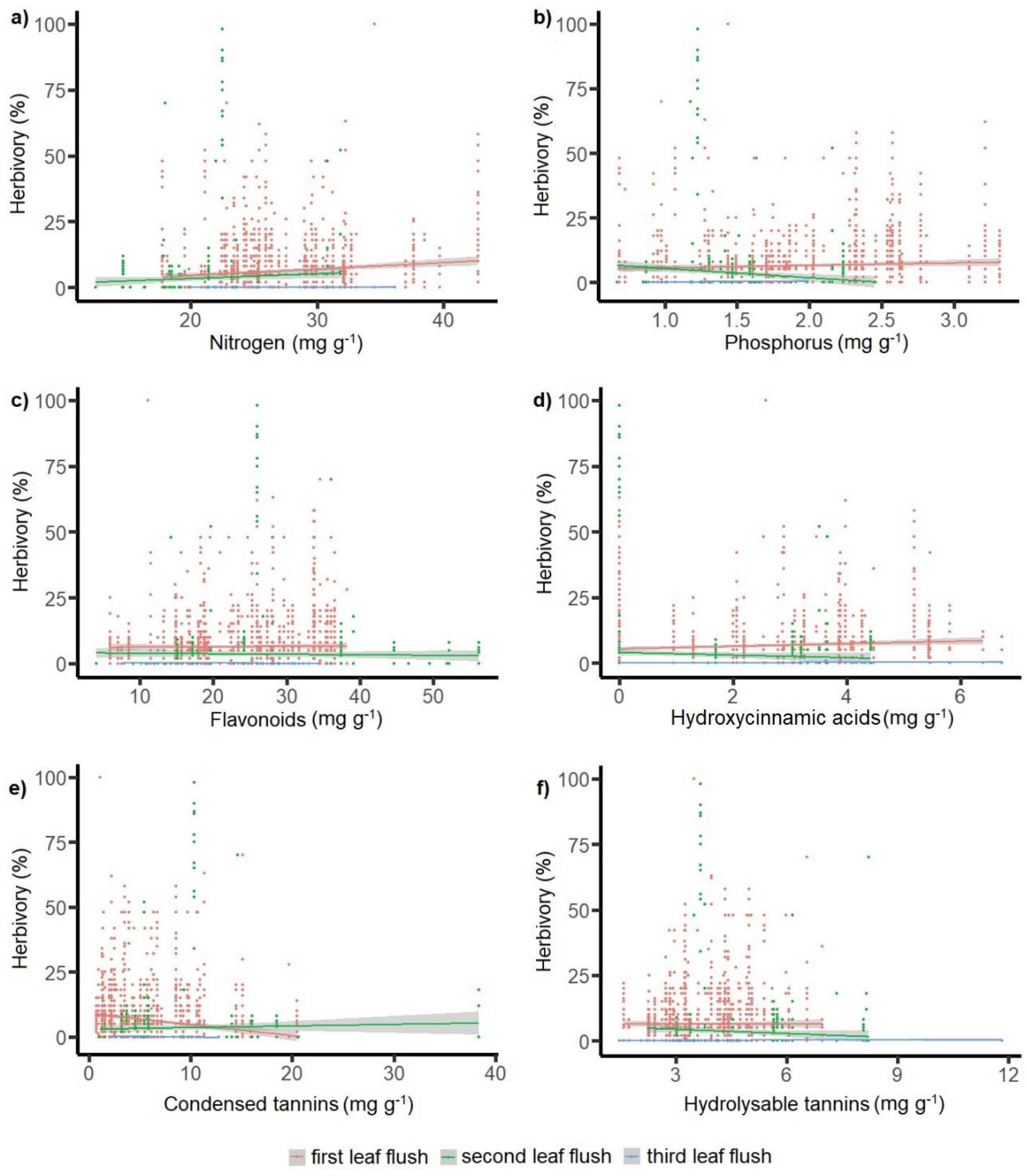

Figure S4. The effect of concentrations of **a)** nitrogen, **b)** phosphorus, **c)** flavonoids, **d)** hydroxycinnamic acids, **e)** condensed tannins and **f)** hydrolysable tannins on the percentage of free-feeding herbivory during the late season in oak leaves (*Quercus robur*). Shown are regression lines with their associated standard error (shaded area), separately for each leaf flush. Concentrations are expressed in mg g−1 of dry tissue.

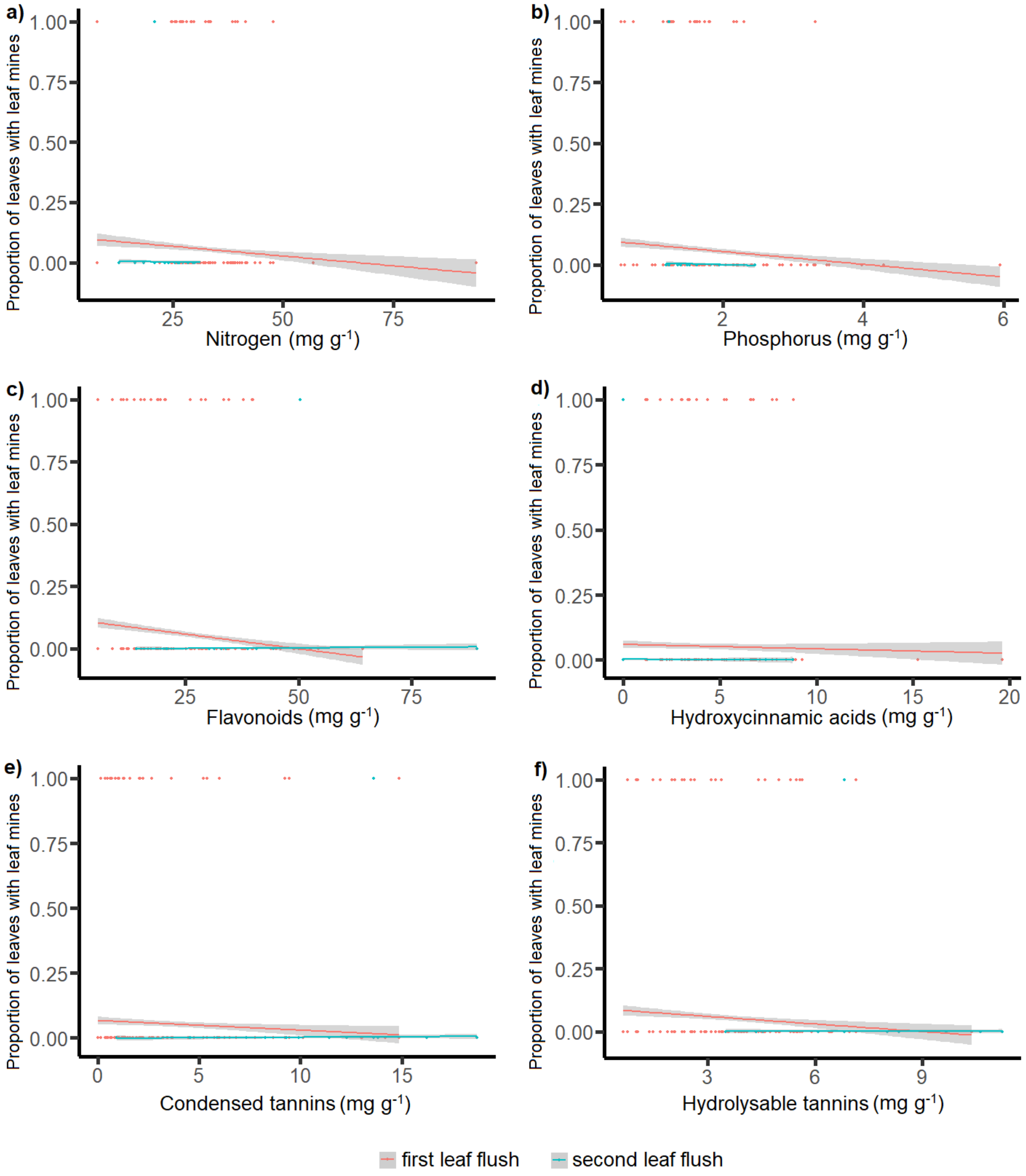

Figure S5. The effect of concentrations of **a)** nitrogen, **b)** phosphorus, **c)** flavonoids, **d)** hydroxycinnamic acids, **e)** condensed tannins and **f)** hydrolysable tannins on the proportion of leaves with leaf mines during the early season in oak leaves (*Quercus robur*). Shown are regression lines with their associated standard error (shaded area), separately for each leaf flush. Concentrations are expressed in mg g−1 of dry tissue.

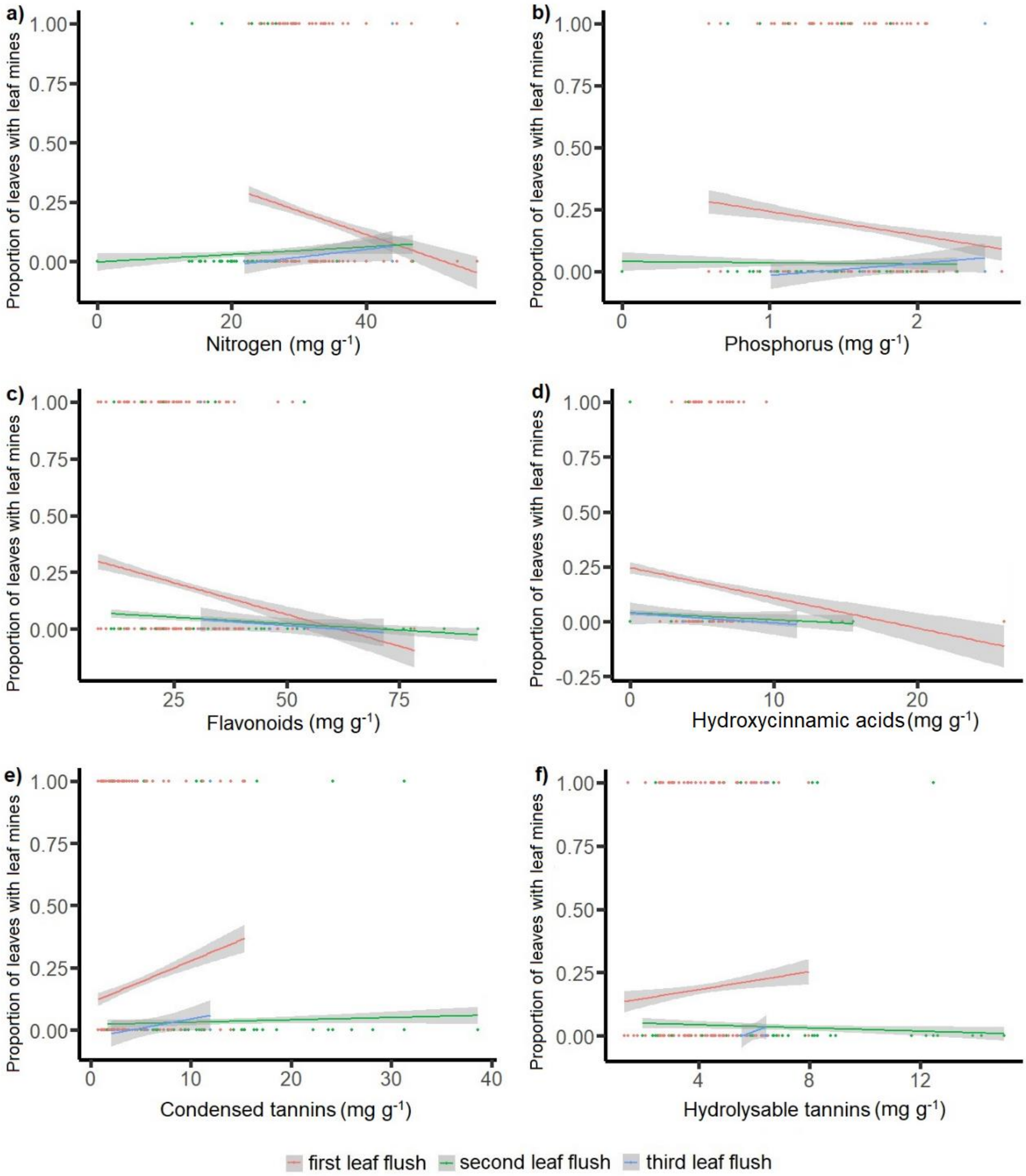

Figure S6. The effect of concentrations of **a)** nitrogen, **b)** phosphorus, **c)** flavonoids, **d)** hydroxycinnamic acids, **e)** condensed tannins and **f)** hydrolysable tannins on the proportion of leaves with leaf mines during the mid season in oak leaves (*Quercus robur*). Shown are regression lines with their associated standard error (shaded area), separately for each leaf flush. Concentrations are expressed in mg g−1 of dry tissue.

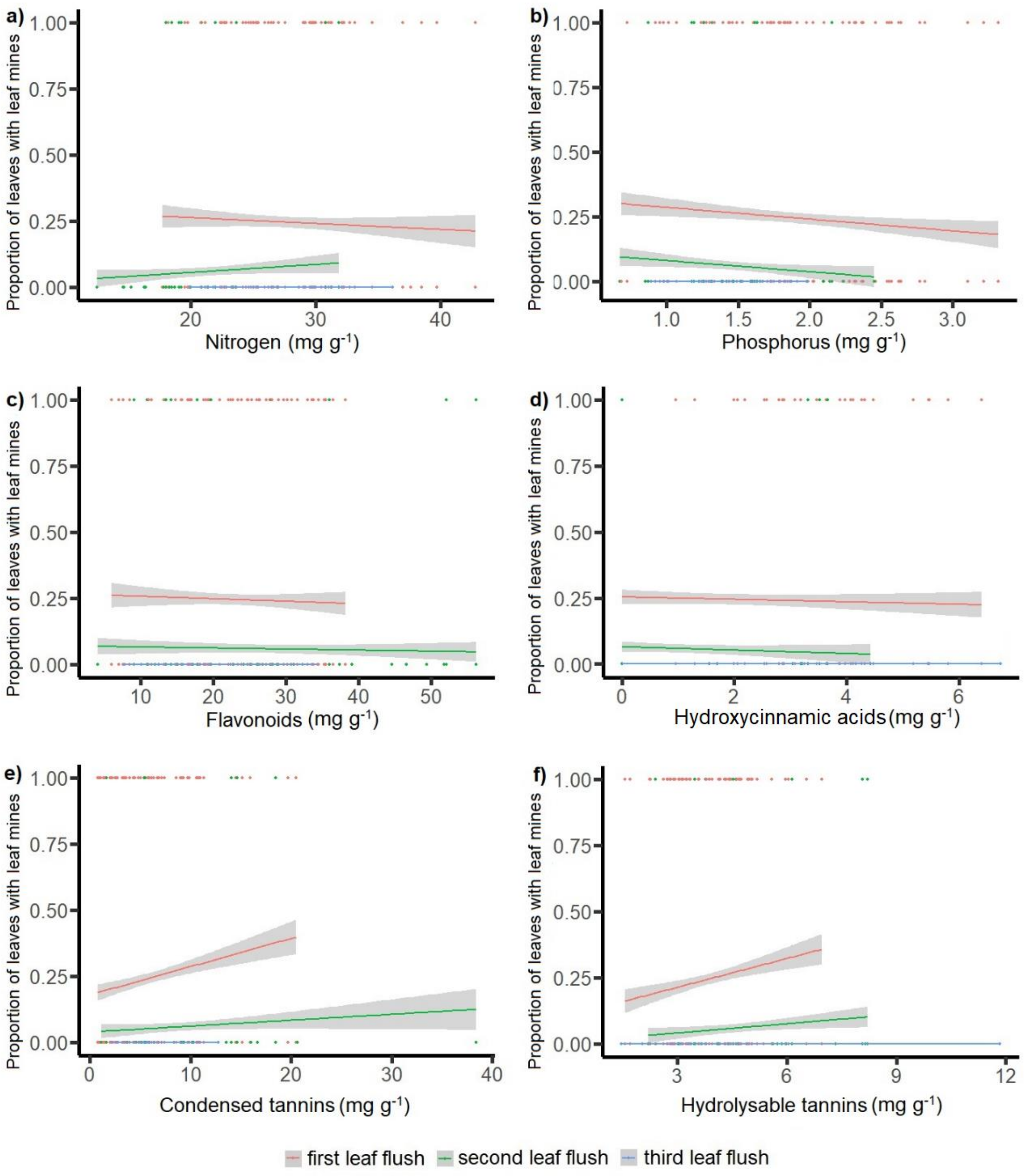

Figure S7. The effect of concentrations of **a)** nitrogen, **b)** phosphorus, **c)** flavonoids, **d)** hydroxycinnamic acids, **e)** condensed tannins and **f)** hydrolysable tannins on the proportion of leaves with leaf mines during the late season in oak leaves (*Quercus robur*). Shown are regression lines with their associated standard error (shaded area), separately for each leaf flush. Concentrations are expressed in mg g−1 of dry tissue.

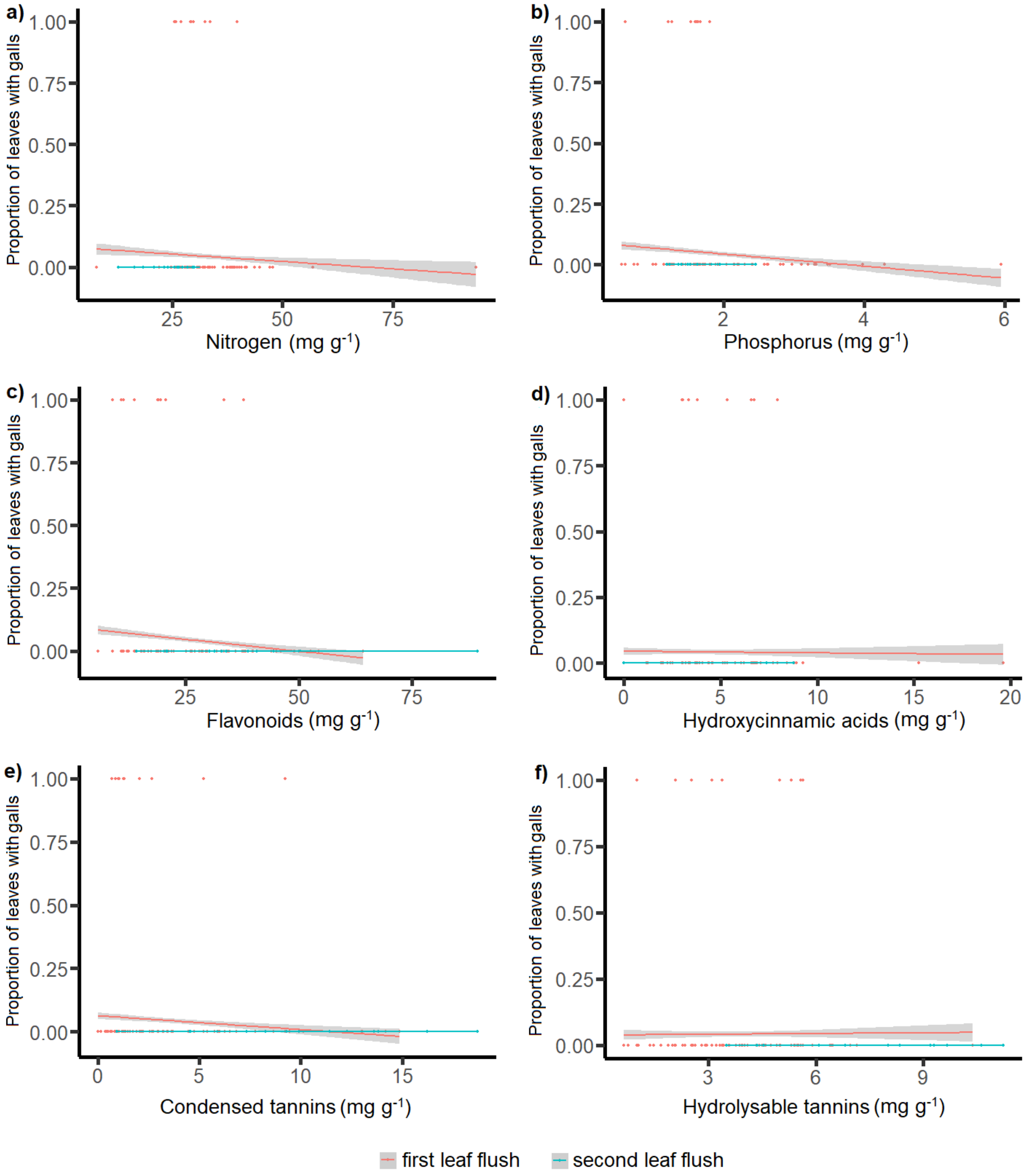

Figure S8. The effect of concentrations of **a)** nitrogen, **b)** phosphorus, **c)** flavonoids, **d)** hydroxycinnamic acids, **e)** condensed tannins and **f)** hydrolysable tannins on the proportion of leaves with galls during the early season in oak leaves (*Quercus robur*). Shown are regression lines with their associated standard error (shaded area), separately for each leaf flush. Concentrations are expressed in mg g−1 of dry tissue.

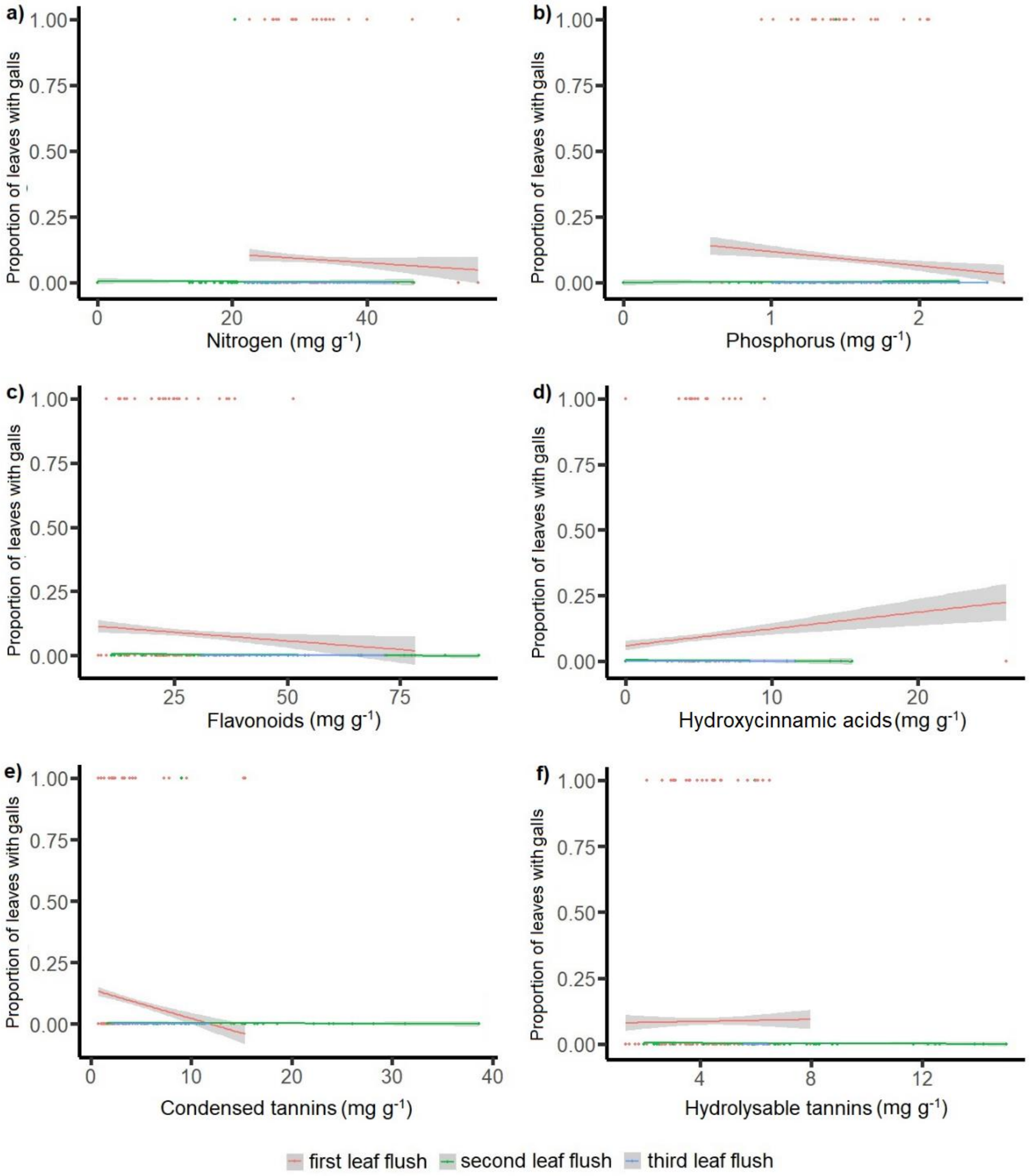

Figure S9. The effect of concentrations of **a)** nitrogen, **b)** phosphorus, **c)** flavonoids, **d)** hydroxycinnamic acids, **e)** condensed tannins and **f)** hydrolysable tannins on the proportion of leaves with galls during the mid season in oak leaves (*Quercus robur*). Shown are regression lines with their associated standard error (shaded area), separately for each leaf flush. Concentrations are expressed in mg g−1 of dry tissue.

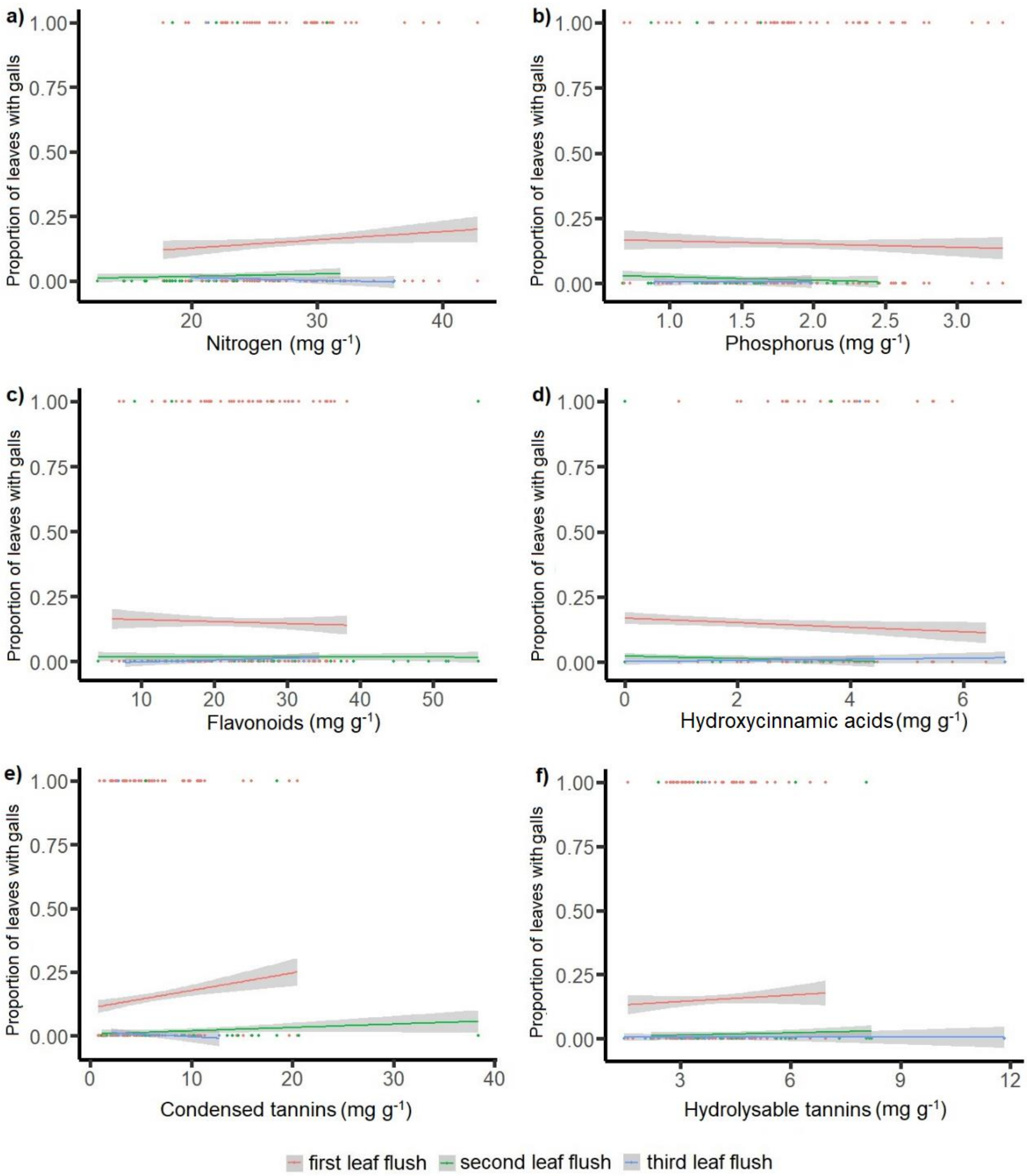

Figure S10. The effect of concentrations of **a)** nitrogen, **b)** phosphorus, **c)** flavonoids, **d)** hydroxycinnamic acids, **e)** condensed tannins and **f)** hydrolysable tannins on the proportion of leaves with galls during the late season in oak leaves (*Quercus robur*). Shown are regression lines with their associated standard error (shaded area), separately for each leaf flush. Concentrations are expressed in mg g−1 of dry tissue.

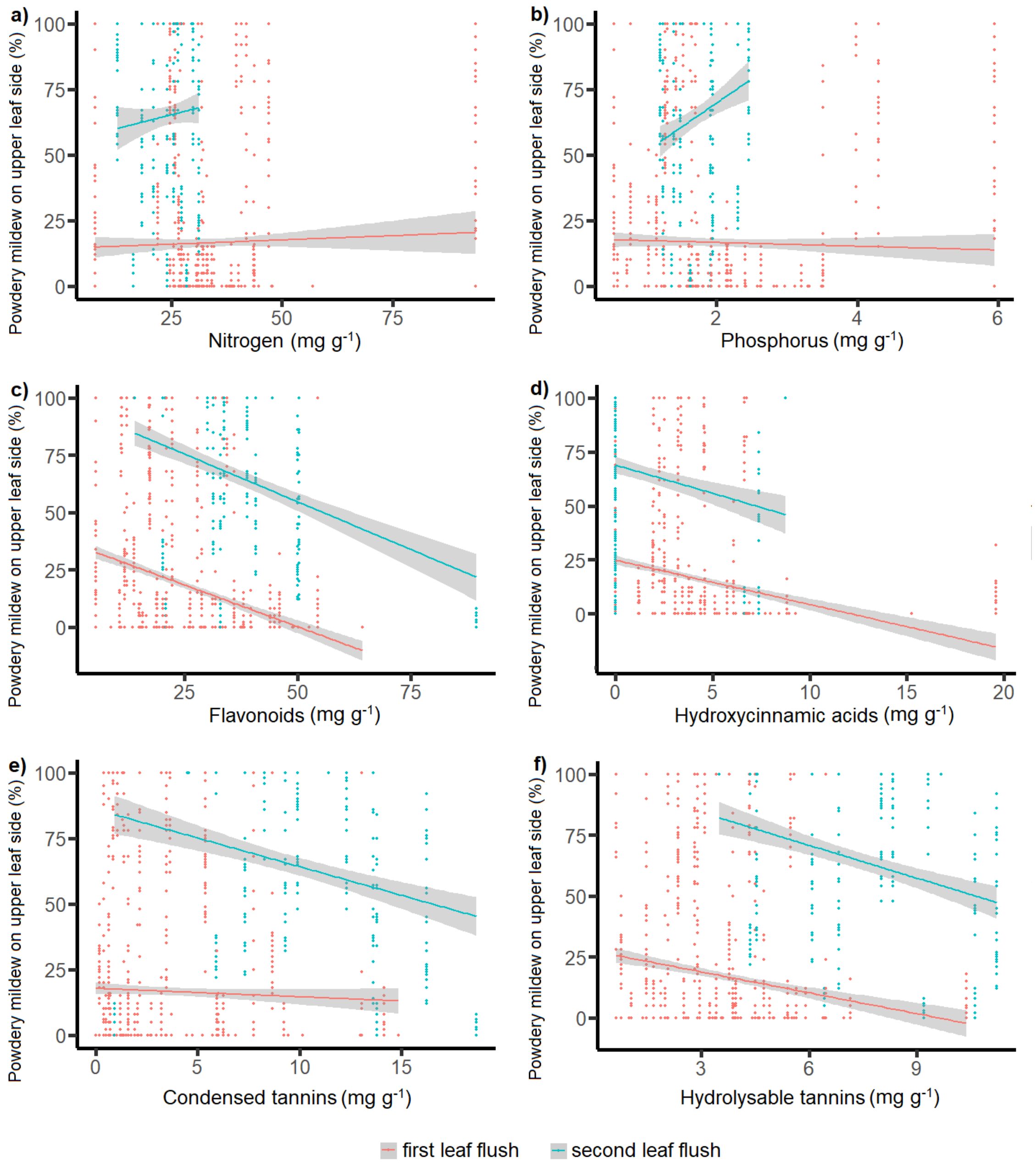

Figure S11. The effect of concentrations of **a)** nitrogen, **b)** phosphorus, **c)** flavonoids, **d)** hydroxycinnamic acids, **e)** condensed tannins and **f)** hydrolysable tannins in infection by oak powdery mildew (*Erysiphe* spp.) on the upper leaf side of the pedunculate oak *Quercus robur* during the early season. Shown are regression lines with their associated standard error (shaded area), separately for each leaf flush. Concentrations are expressed in mg g−1 of dry tissue.

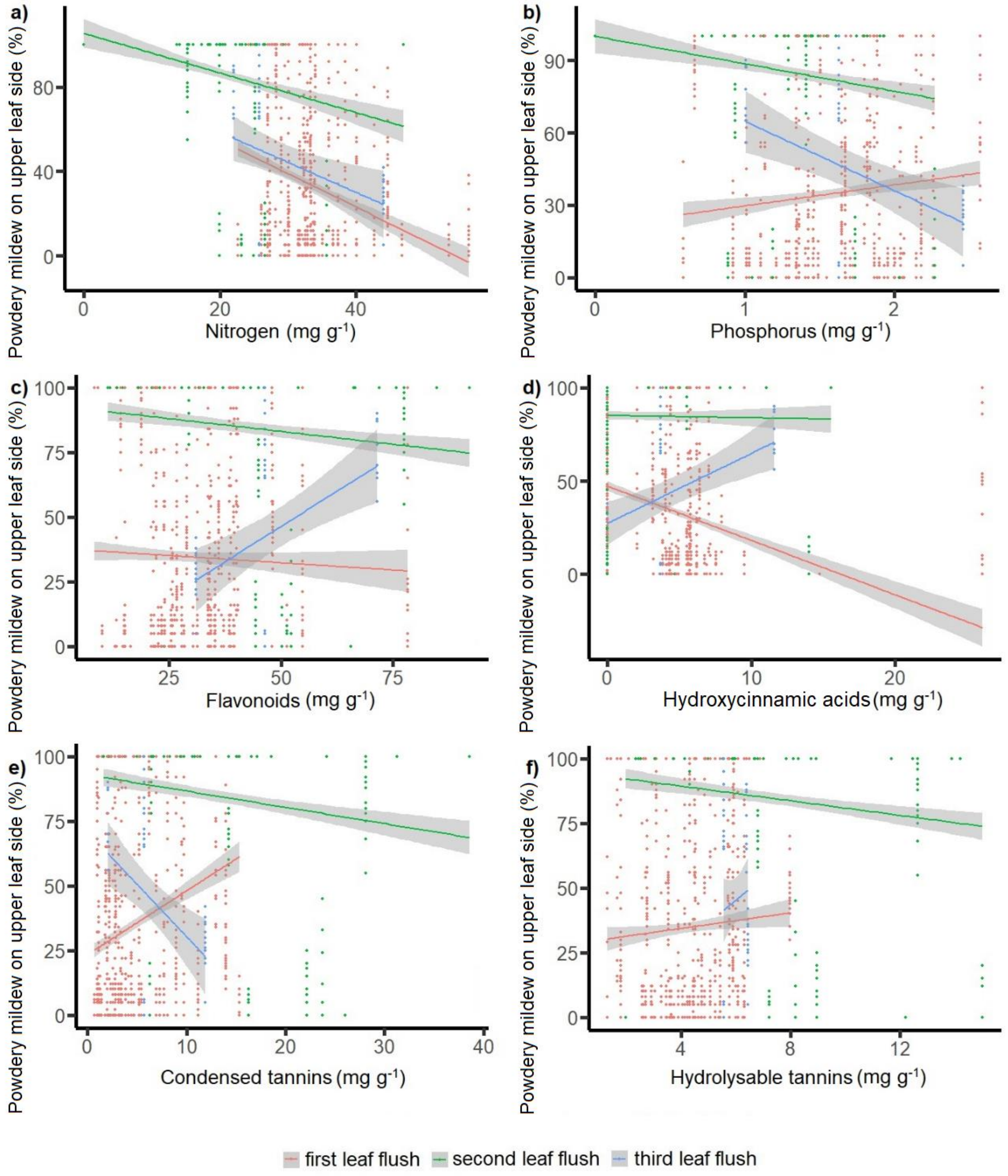

Figure S12. The effect of concentrations of **a)** nitrogen, **b)** phosphorus, **c)** flavonoids, **d)** hydroxycinnamic acids, **e)** condensed tannins and **f)** hydrolysable tannins in infection by oak powdery mildew (*Erysiphe* spp.) on the upper leaf side of the pedunculate oak *Quercus robur* during the mid season. Shown are regression lines with their associated standard error (shaded area), separately for each leaf flush. Concentrations are expressed in mg g−1 of dry tissue.

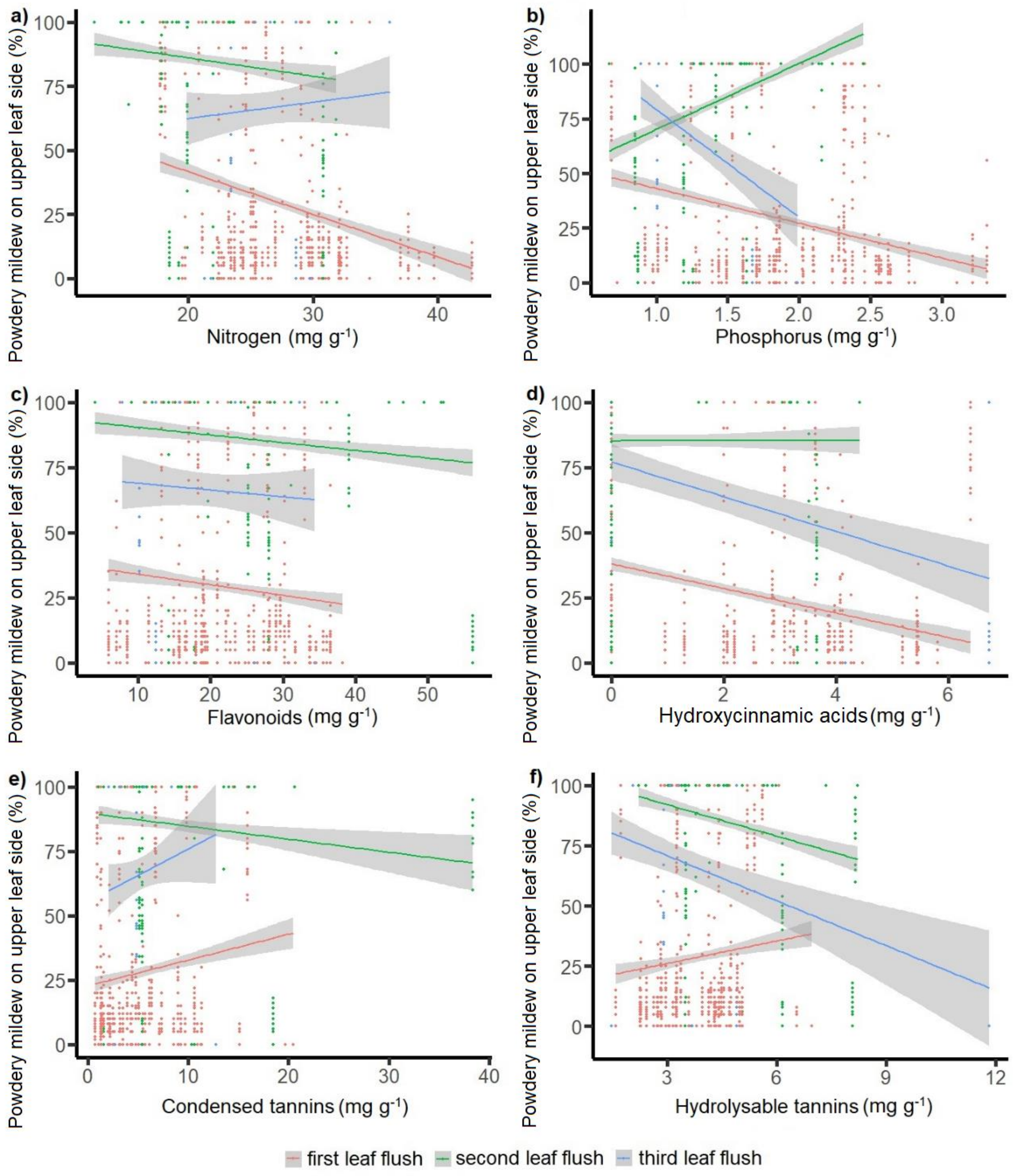

Figure S13. The effect of concentrations of **a)** nitrogen, **b)** phosphorus, **c)** flavonoids, **d)** hydroxycinnamic acids, **e)** condensed tannins and **f)** hydrolysable tannins in infection by oak powdery mildew (*Erysiphe* spp.) on the upper leaf side of the pedunculate oak *Quercus robur* during the late season. Shown are regression lines with their associated standard error (shaded area), separately for each leaf flush. Concentrations are expressed in mg g−1 of dry tissue.

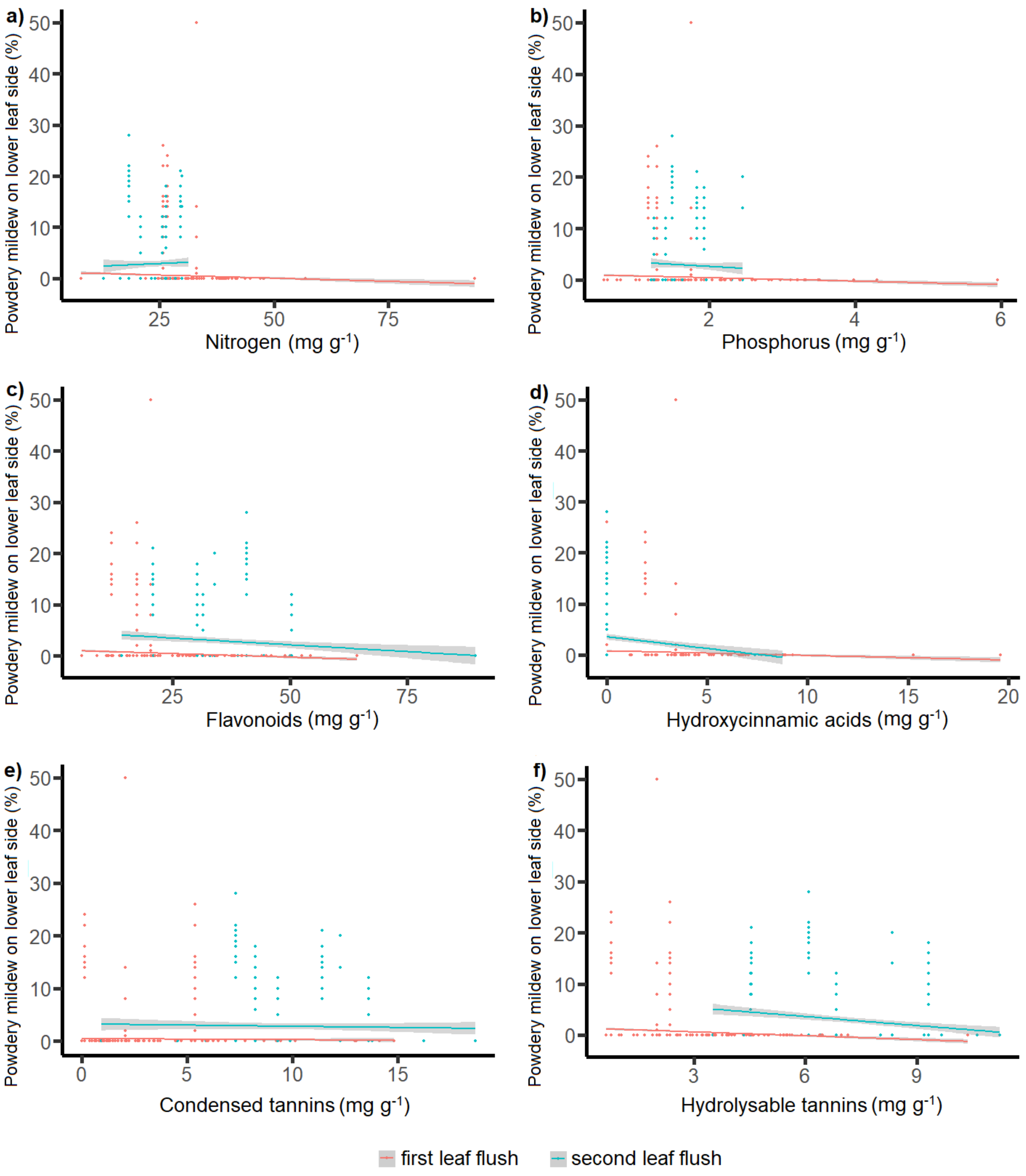

Figure S14. The effect of concentrations of **a)** nitrogen, **b)** phosphorus, **c)** flavonoids, **d)** hydroxycinnamic acids, **e)** condensed tannins and **f)** hydrolysable tannins in infection by oak powdery mildew (*Erysiphe* spp.) on the lower lower leaf side of the pedunculate oak *Quercus robur* during the early season. Shown are regression lines with their associated standard error (shaded area), separately for each leaf flush. Concentrations are expressed in mg g−1 of dry tissue.

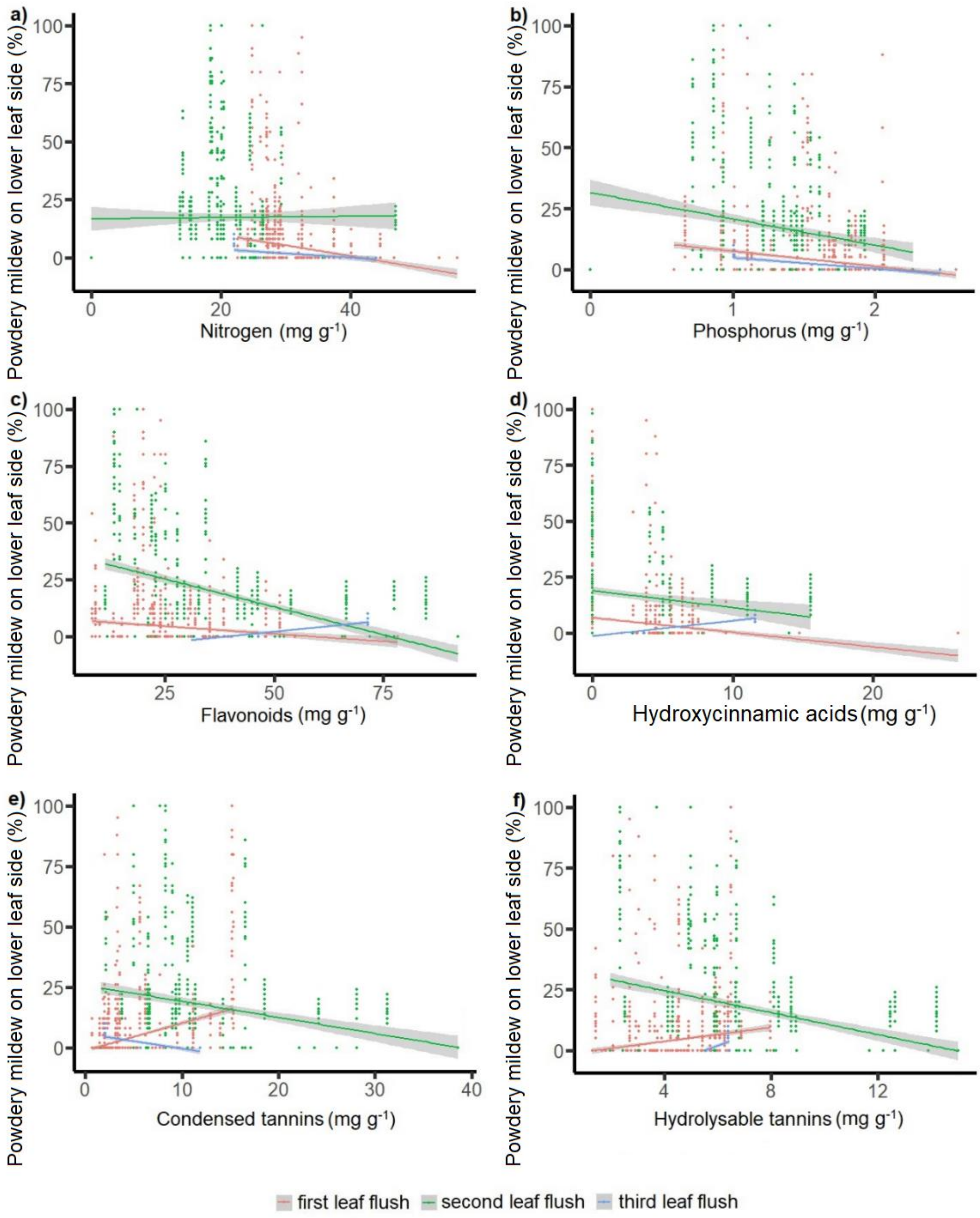

Figure S15. The effect of concentrations of **a)** nitrogen, **b)** phosphorus, **c)** flavonoids, **d)** hydroxycinnamic acids, **e)** condensed tannins and **f)** hydrolysable tannins in infection by oak powdery mildew (*Erysiphe* spp.) on the lower leaf side of the pedunculate oak *Quercus robur* during the mid season. Shown are regression lines with their associated standard error (shaded area), separately for each leaf flush. Concentrations are expressed in mg g−1 of dry tissue.

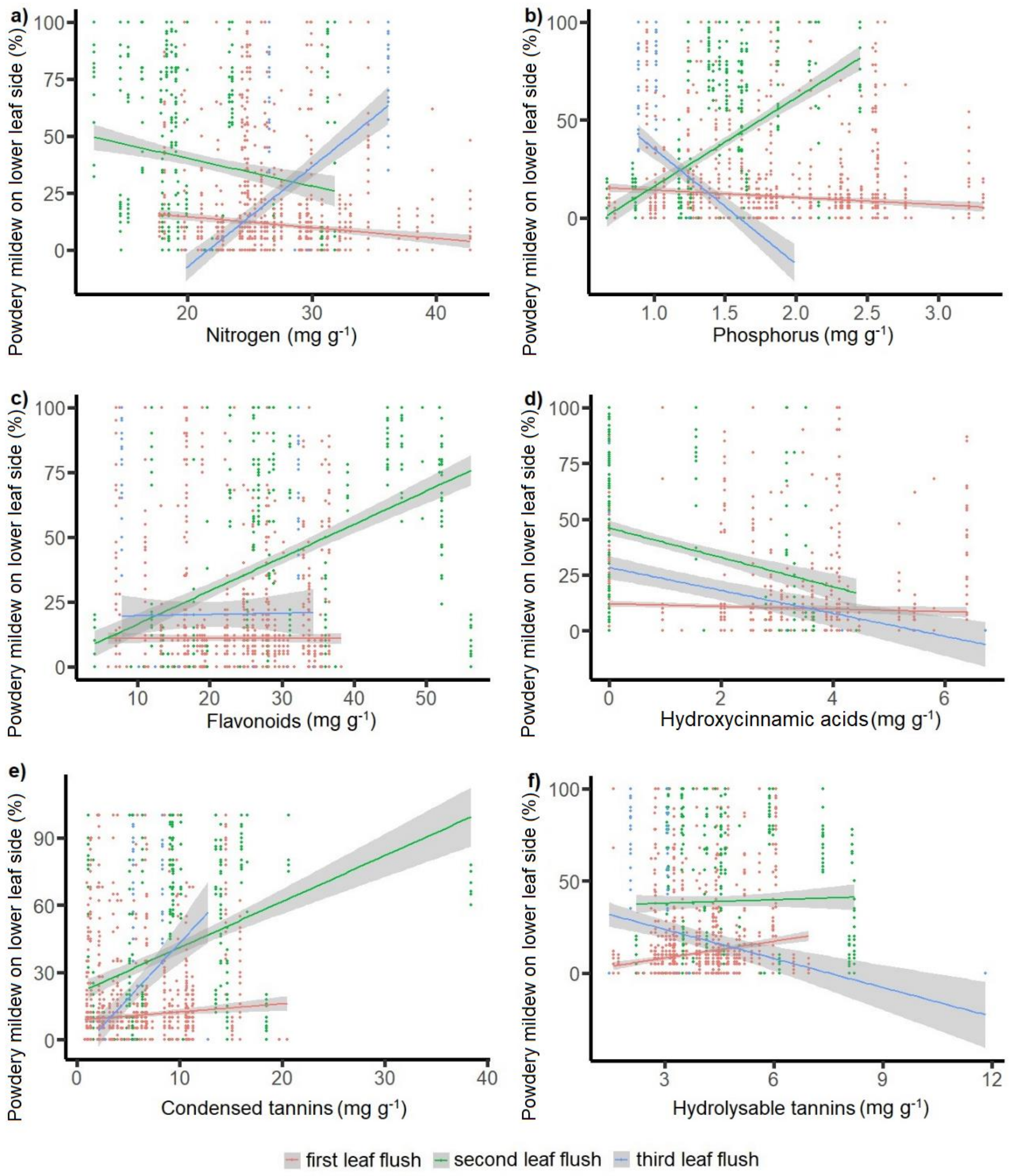

Figure S16. The effect of concentrations of **a)** nitrogen, **b)** phosphorus, **c)** flavonoids, **d)** hydroxycinnamic acids, **e)** condensed tannins and **f)** hydrolysable tannins in infection by oak powdery mildew (*Erysiphe* spp.) on the lower leaf side of the pedunculate oak *Quercus robur* during the late season. Shown are regression lines with their associated standard error (shaded area), separately for each leaf flush. Concentrations are expressed in mg g−1 of dry tissue.

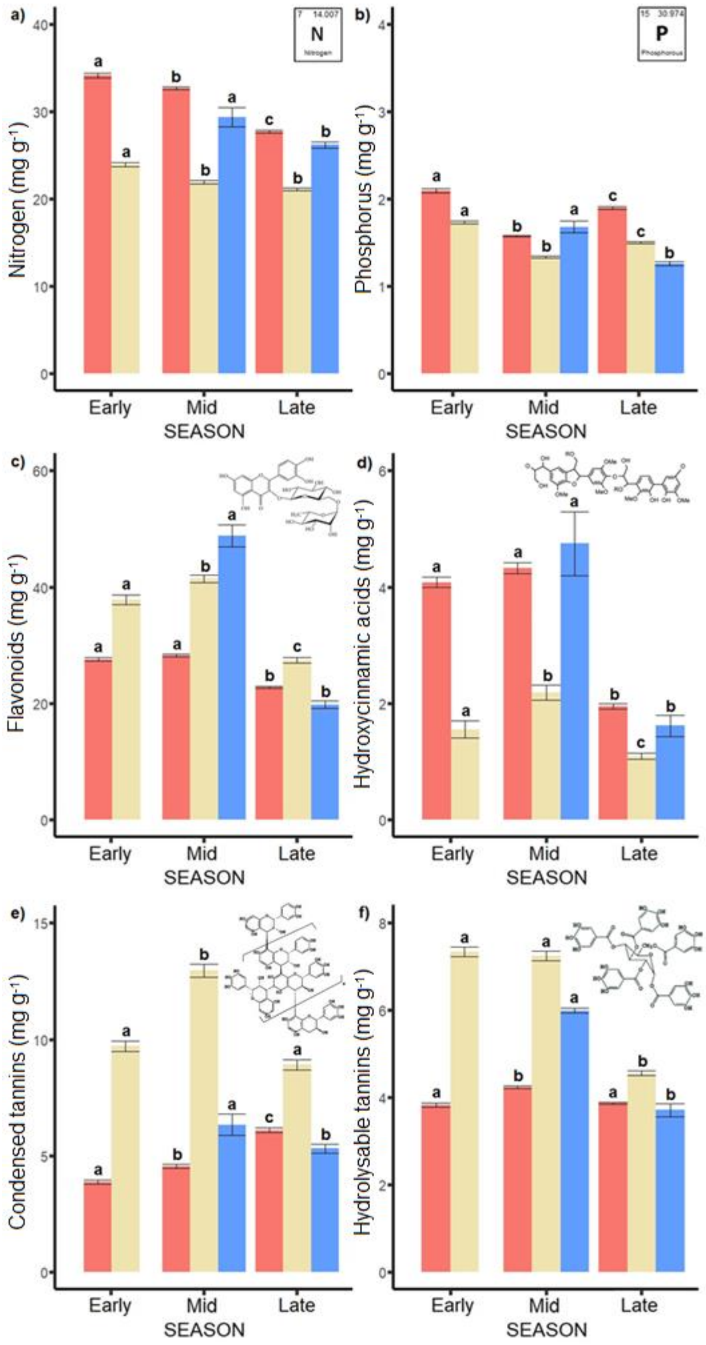

Figure S17. Differences among oak leaf flushes (*Quercus robur*) in the concentration of primary (**a)** nitrogen, **b)** phosphorus) and secondary compounds (**c)** flavonoids, **d)** hydroxycinnamic acids, **e)** condensed tannins and **f)** hydrolysable tannins) during the early, mid and late season. Concentrations are expressed in mg g^{-1} of dry tissue. Bars represent means and error bars represent standard errors. Significant differences within leaf flushes among seasons are indicated by different letters above bars ($p < 0.05$).

NOTES S1: Example of HPLC chromatogram with all the compounds detected.

Table N1. List of compounds detected by HPLC in the example presented in Fig. A1. Shown are the peak identity in Fig. A1, retention time in minutes (RT), experimental mass (molar mass), major ions (MS/MS), detected compound and class of the detected compound.

Peak	RT	Experimental Mass m/z [M-H]	MS/MS (m/z)	Compound	Compound class
1	2.5	191.0567	127, 109, 93, 85	Quinic acid	Hydroxycinnamic acid
2	4.6	457.0565	109, 121, 125, 137, 151, 159, 168, 179, 192, 287, 305,	Epigallocatechin gallate	Condensed tannin
3	5.0	933.0695	915, 871, 613, 569, 493, 489, 467, 340, 300, 249, 125	Castalagin	Hydrolyzable tannin
4	5.3	783.0697	481, 301, 275, 249, 169	Gallotannin	Hydrolyzable tannin
5	5.4	1101.0800	1083, 1039, 995, 915, 613, 577, 569, 425	Ellagitannin (Punicalagin-like)	Hydrolyzable tannin
6	5.5	337.0917	191, 163, 119	Coumaroyl quinic acid	Hydroxycinnamic acid
7	6.2	367.1054	193, 191, 149, 134, 117	Feruloyl quinic acid	Hydroxycinnamic acid
8	6.4	289.0558	109, 121, 123, 125, 137, 151	Epigallocatechin	Condensed tannin
9	6.5	657.1848	367, 193	Feruloyl quinic acid derivative	Hydroxycinnamic acid
10	6.7	463.0885	301	Quercetin hexoside	Flavonoid
11	6.9	599.1054	447, 313, 285, 241, 169	Kaempferol galloyl hexoside derivative	Flavonoid
12	7.2	739.1653	593, 453, 285, 229, 163, 145	Kaempferol coumaroyl derivative	Flavonoid
13	7.5	781.1724	635, 557, 495, 285, 145	Kaempferol derivative	Flavonoid
14	8.2	609.1460	300	Rutin	Flavonoid
15	8.8	823.1921	677, 659, 617, 537, 495, 285, 163, 145	Kaempferol derivative	Flavonoid

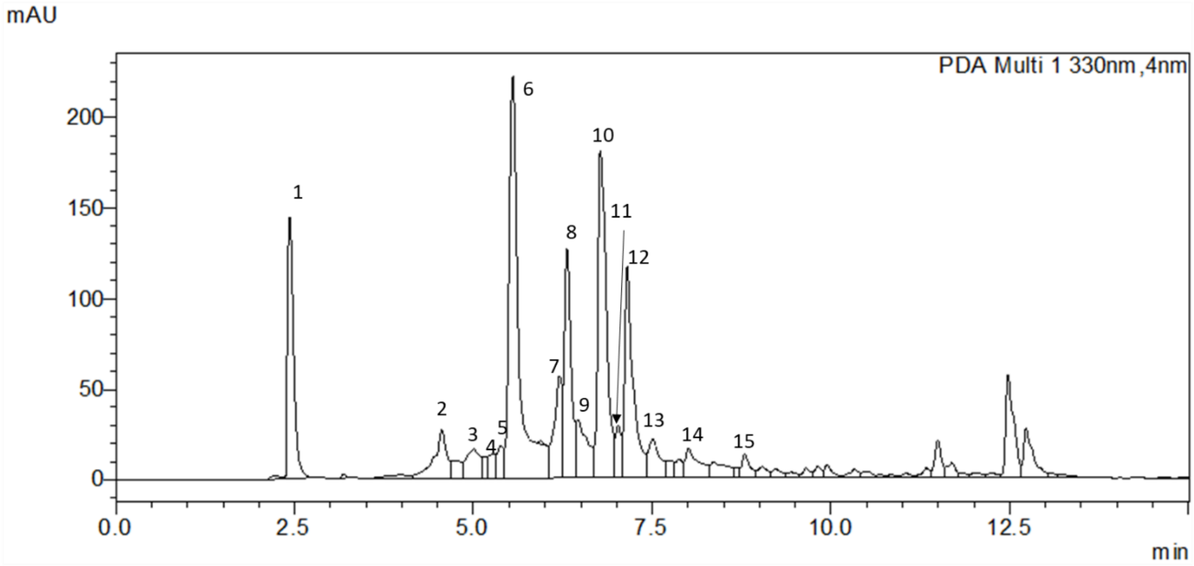

Figure N1. Example of HPLC chromatogram with all the compounds (peaks) detected. The identity of each compound is summarized in Table A1.
